# Therapeutic Cancer Vaccines—Antigen Discovery and Adjuvant Delivery Platforms

**DOI:** 10.3390/pharmaceutics14071448

**Published:** 2022-07-11

**Authors:** Neftali Ortega Alarcon, Maddy Jaramillo, Heidi M. Mansour, Bo Sun

**Affiliations:** 1 Skaggs Pharmaceutical Sciences Center, College of Pharmacy, The University of Arizona, Tucson, AZ 85721, USA; n.ortega@mvtpharma.com (N.O.A.); mrjaramillo@pharmacy.arizona.edu (M.J.); hmansour@fiu.edu (H.M.M.); 2 The University of Arizona Cancer Center, Tucson, AZ 85721, USA; 3 Department of Medicine, College of Medicine, The University of Arizona, Tucson, AZ 85724, USA; 4 BIO5 Institute, The University of Arizona, Tucson, AZ 85721, USA

**Keywords:** therapeutic vaccine, tumor antigens, adjuvants, delivery systems, combination therapies

## Abstract

For decades, vaccines have played a significant role in protecting public and personal health against infectious diseases and proved their great potential in battling cancers as well. This review focused on the current progress of therapeutic subunit vaccines for cancer immunotherapy. Antigens and adjuvants are key components of vaccine formulations. We summarized several classes of tumor antigens and bioinformatic approaches of identification of tumor neoantigens. Pattern recognition receptor (PRR)-targeting adjuvants and their targeted delivery platforms have been extensively discussed. In addition, we emphasized the interplay between multiple adjuvants and their combined delivery for cancer immunotherapy.

## 1. Introduction

Vaccines have been a necessary fixture in modern society for the promotion and perpetuation of public and personal health. Vaccines achieve this by inducing an adaptive immune response (via an antigen and adjuvant combination) that will recognize, target, and eliminate invading pathogens in the infected host [1]. This discovery was made by Edward Jenner in the late 1700s when trying to treat smallpox, and these fundamental principles are still being used to treat a wide array of diseases with known pathogenic etiologies [1,2]. Nowadays, vaccines have proved their significance in battling against infectious diseases such as coronavirus disease [3], human immunodeficiency virus infection [4], and cancers as well [5]. This review focused on the basic and translational research in therapeutic subunit vaccines for cancer immunotherapy. Antigens and adjuvants are key components of subunit vaccines. We summarized several classes of tumor antigens and biochemical/bioinformatic approaches to identify novel tumor antigens. Recent advances in PRR-targeting adjuvants and their targeted delivery platforms have been extensively discussed. In addition, we emphasized the interplay among multiple adjuvants and their combined delivery for cancer immunotherapy.

## 2. Tumor Antigens for Cancer Vaccines

The principles of vaccine development, however, eluded the grasp of researchers in the context of cancer immunotherapy until 1957, when Prehn and Main showed that an immune response could be induced in mice against carcinogen-induced sarcomas, which also prevented mice from developing tumors when further challenged with the same tumor cells [6]. This was further solidified in 1991 when van der Burrgen and colleagues discovered the tumor antigen encoding gene MZ2-E through complimentary DNA (cDNA) transfections in cells with relevant major histocompatibility complexes (MHC). After this, relevant transfectants were able to be identified by anti-tumor cytotoxic T-lymphocytes (CTLs) [7]. Since then, a great number of advances have been made in the discovery of novel tumor antigens.

MHCs are the key components of the adaptive immune system that recognize foreign proteins. They are expressed on the surface of most nucleated non-immune and immune cells and present antigenic peptide fragments to either CD8^+^ or CD4^+^ T-cells for an adaptive immune response [8,9,10]. The general structure of an MHC is composed of immunoglobulin-like anchoring peptides, which fixes the MHC to the exterior of cellular membrane and a peptide-binding region (PBR), which is responsible for antigen recognition and presentation to the T-cell receptors (TCR). MHC molecules are further subcategorized into class I and class II molecules [8,9,10,11,12]. MHC class I molecules are heterodimeric molecules that are made up of two polypeptide chains: an α chain that is comprised of three domains (α1, α2, α3) and a smaller β_2_-microglobulin chain [13]. The α1 and α2 domains are key components of the PBR on MHC I and their inherent polymorphisms mitigate and influence antigenic peptides binding to the PBR for antigen presentation to CD8^+^ CTLs [9,12,13]. MHC class I molecules are consistently expressed on the surfaces of most nucleated cells except for sperm cells and select neuronal cells. MHC class II molecules are also heterodimeric molecules that are composed of an α chain and a β chain; however, they have two distinct domains referred to as α1, α2 and β1, β2 [8,12]. The PBR in MHC II is composed of α1 and β1 domains [8,10,12]. MHC class II molecules are only expressed on antigen presenting cells (APCs) such as dendritic cells (DCs), macrophages, and B-cells, which specifically present to CD4^+^ T cells for an immune response [10,12,14]. 

If a tumor antigen was presented restrictively on an MHC class I molecule to an appropriately matched TCR on a CD8^+^ CTL, then cytolytic actions will be carried out by CTLs, leading to the shrinkage or elimination of the tumor and an established immunity against the same tumor antigens [15,16,17]. Cytolytic activity is also seen when tumor antigens are presented restrictively on MHC class II molecules for tandem antigen recognition between CD4^+^ T-cell TCR and MHC class II molecule [18,19,20]. However, this is not the main anti-tumor effect enacted by CD4^+^ T-cells but a more assistive role observed via the activation of immune effector pathways that stimulates cytokine production and other aspects of the innate immune system [21,22]. 

### 2.1. Classification of Tumor Antigens 

**Figure 1:**
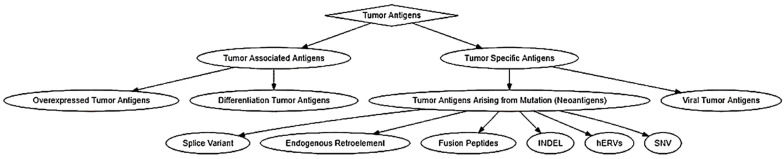
Tumor antigen classifications.

Tumor antigens were classified into two categories: tumor-associated antigens (TAAs) and tumor-specific antigens (TSAs) (Figure 1). TAAs are tumor antigens that are expressed in normal germline cells as well as tumor cells [23]. Due to the wide expression profile of TAAs, they have been further subcategorized into differentiation tumor antigens and overexpressed tumor antigens [23]. Overexpressed tumor antigens are a class of TAAs that could be found in normal tissues but are expressed at an elevated level in various cancerous tissues [23,24]. A well-known example is human epidermal growth factor receptor 2 (also known as HER2 or ERBB2). Differentiation tumor antigens are a class of TAAs that are limited in their expression to one tissue type and show lineage-specific expression. These antigens are expressed in tumors and normal cells that are derived from the same cellular origin [24,25]. Antigens derived from melanocyte differentiation proteins are typical differentiation antigens that are expressed in melanomas but can be expressed in normal skin melanocytes and retinal tissue [25,26]. Due to the “self” nature of these antigens, there will be a propensity for the development of autoimmune disorders, such as the occurrence of vitiligo after the chemoimmunotherapy of metastatic melanoma [27]. More examples of TAAs and TSAs were summarized in the supplementary tables.

TSAs are cancer antigens that are specifically expressed in malignant cancer cells and have a wide variety of expression among different cancer types. For example, the expression of cancer/testis antigens are restricted to the testis and ovary in normal tissues, but they could be found in a wide range of human tumors [28,29]. Neoantigens are tumor antigens that are only expressed in tumor cells and usually arise from non-synonymous single-nucleotide variant mutations (SNVs), but non-mutated neoantigens have also been identified [8,23,30]. Tumor antigens arising from mutations are TSAs that are expressed ubiquitously across all cell types but are mutated when they are expressed in tumors. These mutations could arise from SNV, insertion and deletion (INDEL), gene fusion, splice variant, endogenous retroelement, and human endogenous retrovirus mutations (hERVs) which induce a change in the amino acid sequence of a protein [24,25,31,32,33,34]. These changes in the amino acid sequences further differentiate a protein from its normal expression profile in non-cancerous cells, subsequently allowing the generation of novel peptide epitopes that can then participate in restrictive presentation on MHC molecules for an adaptive immune response [24,25]. For example, the transcript for the BCR-ABL fusion protein is formed via reciprocal translocation between chromosomes 9 and 22 [35]. The chromosomal abnormality was first identified in cases of chronic myeloid leukemia (CML) [36]. BCR-ABL has been shown to be expressed in more than 95% of CML cases [37], but it is also present in approximately 10% to 20% of adults and 2% to 5% of children with acute lymphoblastic leukemia (ALL) [38,39,40], and in some cases of acute myeloid leukemia (AML) [41,42,43], lymphomas [44,45,46], and myelomas [47,48,49]. Besides, K-ras protein is found to be highly mutated in cancers with the bulk of the mutations occurring at position 12 which is termed KRAS G12D, with the change from glycine (G) to aspartic acid (D) or valine (V) [50]. Table 1 provides a summary of neoantigens and their HLA classes. Antigens derived from oncogenic virus comprise another relatively small but indispensable member of the TSA family [24]. The best examples of viral tumor antigens are E6 and E7 oncogenic proteins derived from human papillomavirus 16 (HPV-16). The E6 oncogenic protein is capable of producing epitopes for restrictive presentation on the class-I HLA molecule HLA-B18 for MHC-I [51]. The E7 oncogenic protein yielded an epitope capable of binding in a restrictive fashion on the class-I HLA molecules HLA-A2 and HLA-B18 for MHC-I and on the class-II HLA molecule HLA-DQ2 for MHC-II [51,52,53].

### 2.2. Biochemical and Bioinformatic Approaches for the Identification of Tumor Antigens

#### 2.2.1. Serological Analysis of Recombinant Tumor cDNA Libraries (SEREX)

SEREX is a technique developed by Sahin, Pfreundschuh et al., whereby one can identify novel tumor antigens through the sampling of mRNA from fresh tumor specimens, instead of in vitro cancer cell lines [106]. This is because in vitro cancer cell lines can be subject to either a loss or unwanted generation of cancer antigens, due to mutations that might arise during the continuance of the cell culture. The mRNA extracted from the fresh tumor specimen is used to build a cDNA library and subsequently cloned into a λ phage expression vector. This λ phage expression vector is transfected into *Escherichia coli* for the recombinant expression of potential cancer antigens in the cDNA library. Recombinant proteins are collected and transferred onto a nitrocellulose membrane, blocked, and exposed to autologous diluted serum (1:100 or 1:1000) from the same patient that tumor specimens are taken from. The serum is diluted to ensure that only high-titer IgG antibodies react with the recombinant proteins on the nitrocellulose membrane. Subsequently, a secondary immunoscreening is performed with anti-human IgG for the purification and identification of positive clones while eliminating the false positives that can arise from residual recombinant immunoglobulin (IgG) expression, due to the B-cells and plasma cells that are present in the tumor specimen being sampled. Finally, positive clones are subcloned for isolation of that specific antigenic cDNA fragment and that cDNA fragment is sequenced to determine its nucleotide sequence [107,108,109]. One of the major drawbacks of SEREX is that the bacteria is incapable of expressing low abundant TAAs and their tumor-specific post-translational modification [110,111]. SEREX-defined antigens are usually weakly immunogenic due to the lack of mutations or structural aberrance [112]. SEREX has also been criticized for its demanding protocol and poor reproducibility [111].

#### 2.2.2. Computational Prediction Methods for Cancer Antigens

**Figure 2:**
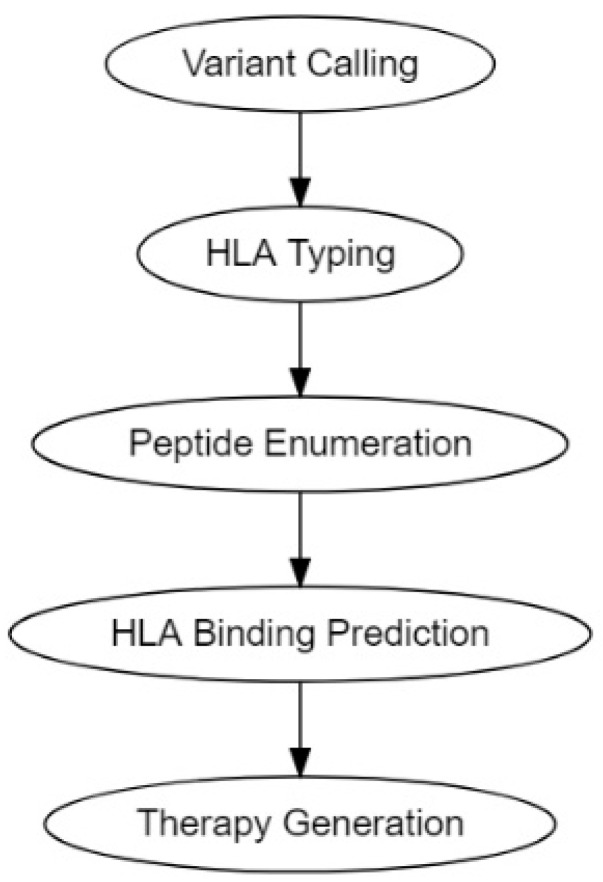
Tumor antigen computational pipelines.

The traditional pipeline for computational tumor-specific antigen prediction segments itself into five distinct steps: variant calling, HLA typing, peptide enumeration, HLA binding prediction, and finally therapy generation (Figure 2) [30,113]. Variant calling involves predicting potential cancer antigens through methods that use data from high-throughput genetic sequencing (RNA-Seq or DNA-Seq). This genetic data is processed through algorithms best suited to predict the potential antigenicity of a TSA, depending on its mutational origin: SNV, INDEL, frameshifts, fusion proteins, endogenous retroelement, or hERVs [30,113,114]. HLA typing is performed to determine HLA allele frequencies [30,113,115]. Peptide enumeration is done to determine the peptide sequences of the potential antigenic mutants and to sort them from incompatible antigenic sequences that arise from non-sense mutations and other non-functional genetic aberrations [30,113,116]. HLA binding prediction is enacted to determine the binding of affinity of the antigenic peptide to the corresponding HLA molecule. This HLA-to-peptide affinity quantification is usually expressed as either a ranked percentile or a K_D_ ≤ 500 nM (standard cutoff for detection) [80,117]. Finally, the genetic information gathered was utilized to make vaccine and cellular therapeutics such as DNA, RNA, peptide, and autologous DC or T-cell vaccines. 

Computational prediction pipelines such as Epidisco (ver.1.0, lex Rubinsteyn, et al.), Antigen.garnish (ver.2.3.1, Andrew J Rech, Lee Richman), pVACtools (ver.3.0.2, Jasreet Hundal, et al.), Neopepsee (ver.3.0.1, Sora Kim), MuPeXI (ver.1.2.0, Anne-Mette Bjerregaard, Aron C. Eklund), TSNAD (ver.2.0.1, Zhan Zhou et al.), NeoepitopePred (ver.1.0, Jinghui Zhang et al.), and INTEGRATE-Neo (ver.1.2.1, Jin Zhang, et al.) condense these steps (variant calling, HLA typing, peptide enumeration, HLA binding prediction) into succinct computational workflows [118,119,120,121,122,123,124,125]. Epidisco is very versatile in the variety of TSAs it can predict. It specializes in identifying potential TSA neoantigens of SNV, INDEL, splice variant, and gene fusion mutational origins that bind MHC class I molecules [123]. Antigen.garnish and pVACtools also have extensive predictive abilities, with both being able to predict TSA neoantigens of SNV, INDEL, and gene fusion mutational origins [120,122]. However, unlike Epidisco and pVACtools, which only allow for MHC-I antigen prediction, Antigen.garnish can do both MHC class I and class II binding predictions for the neoantigens it predicts [120,122,123]. MuPeXI, TSNAD, and Neopepsee are all used to predict TSAs that arise from SNV and INDEL mutational origins [118,121,125]. Even though MuPeXI, TSNAD, and Neopepsee all work to call the same mutational variants (SNV and INDEL); Neopepsee can only make binding predictions for MHC-I whereas MuPeXI and TSNAD can make binding predictions for both MHC-I and MHC-II [118,121,125]. NeoepitopePred works to predict SNV, and gene fusion TSA mutational variants and can make MHC-peptide binding predictions for MHC-I [119]. INTEGRATE-Neo is a very specialized workflow in that it only predicts TSA mutational variants of gene fusion origins for binding to MHC class I molecules [124]. Although there are currently no programing pipe lines in place that specialize in the MHC binding prediction for TSAs originating from retroelements or human endogenous retroviruses (hERVs), RepeatMasker and hervQuant are variant calling tools (not complete computational pipelines) that can be used to identify potential TSAs originating from retroelements and hERVs respectively [126,127].

### 2.3. Delivery of Neoantigens 

Neoantigens can be delivered in the form of synthetic long peptides or neoepitope-encoding mRNA or DNA [5]. Direct injection of soluble subunit antigens and immune adjuvants can only induce modest immune responses due to their uncontrolled systemic distribution and poor targeting and accumulation in lymphoid organs. DNA requires electroporation-facilitated delivery and extra processing before presentation by DCs [5,128]. mRNA needs delivery platforms to facilitate the intracellular delivery and protect it from ribonuclease degradation [129,130]. Therefore, neoantigen vaccines formulated with novel technologies and biomaterials, such as lipids and biodegradable polymers, are pursued to improve the safety and efficiency of neoantigen delivery. Current progress in the delivery of neoantigen vaccines has been discussed in other reviews [131,132].

## 3. Vaccine Adjuvants 

Adjuvants are known as a variety of substances used in combination with a specific antigen that produce stronger immunity than the antigen alone [133]. Incorporating an adjuvant in a vaccine does not only strengthen the adaptive response to antigens but also enables a comparable response with a lower dose of antigens or less frequent vaccinations relative to unadjuvanted vaccines [134,135]. As such, adjuvants have become essential components of many successful vaccines and those still in clinical trials. For example, the adjuvant system 04 (AS04), consisting of aluminum salt particles loaded with MPL (3-*O*-desacyl-4′-monophosphoryl lipid A), has been used in two licensed vaccines, Cervarix^TM^ against human papillomavirus (HPV) and Fendrix^TM^ against hepatitis B virus [136,137]. Poly ICLC is a derivative of toll-like receptor (TLR) 3 agonist polyriboinosinic-polyribocytidylic acid (poly(I:C)) stabilized with carboxymethylcellulose and poly-L-lysine [138], which has been widely utilized as an adjuvant in therapeutic vaccines against different cancers in more than 50 clinical trials [139]. Several clinically tested vaccine adjuvants were summarized in Table 2. 

**Figure 3:**
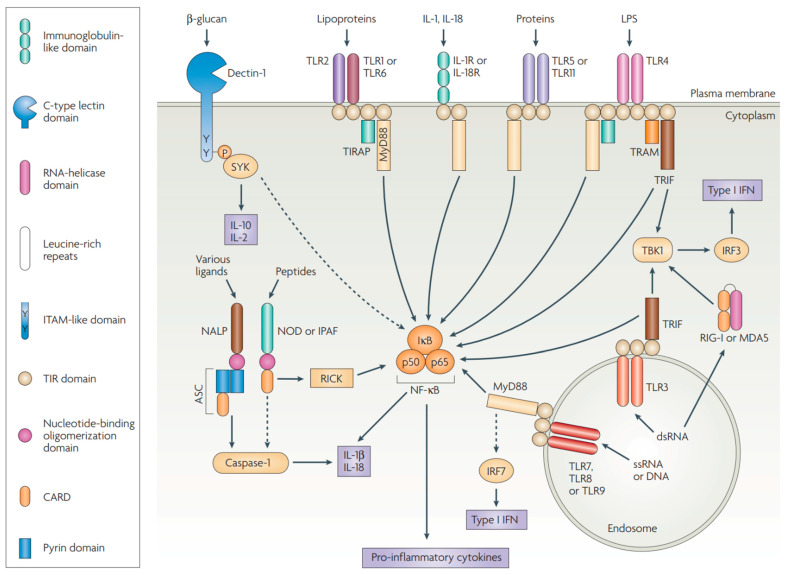
Schematic representation of the structure and main signaling pathways of the PRR families. Only the adaptor molecules and the main signaling pathways that differentiate the different classes of PRR are shown. Dectin-1 (a β-glucan receptor) is shown as an example of various cell-surface PRRs, some belonging to the lectin-like family, and some linked with the immunoreceptor tyrosine-based activation motif (ITAM)-containing adaptor Fc receptor γ-chain (FcRγ), the activation of which can markedly affect TLR signaling. ASC, apoptosis-associated speck-like protein containing a CARD (caspase-recruitment domain); ds, double-stranded; IFN, interferon; IκB, inhibitor of NF-κB; IL, interleukin; IPAF, ICE-protease-activating factor; IRF, IFN-regulatory factor; LPS, lipopolysaccharide; MDA5, melanoma-differentiation-associated gene 5; MyD88, myeloid differentiation primary-response gene 88; NALP, NACHT-, LRR- and pyrin-domain-containing protein; NOD, nucleotide-binding oligomerization domain; RICK, receptor-interacting serine/threonine kinase; RIG-I, retinoic-acid-inducible gene I; ss, single-stranded; TBK1, TANK-binding kinase 1; TIRAP, Toll/IL-1R (TIR)-domain-containing adaptor protein; TRAM, TRIF-related adaptor molecule; TRIF, TIR-domain-containing adaptor protein inducing IFNβ; SYK, spleen tyrosine kinase. Adapted with permission from [149]. Copyright 2007, Springer Nature.

**Figure 4:**
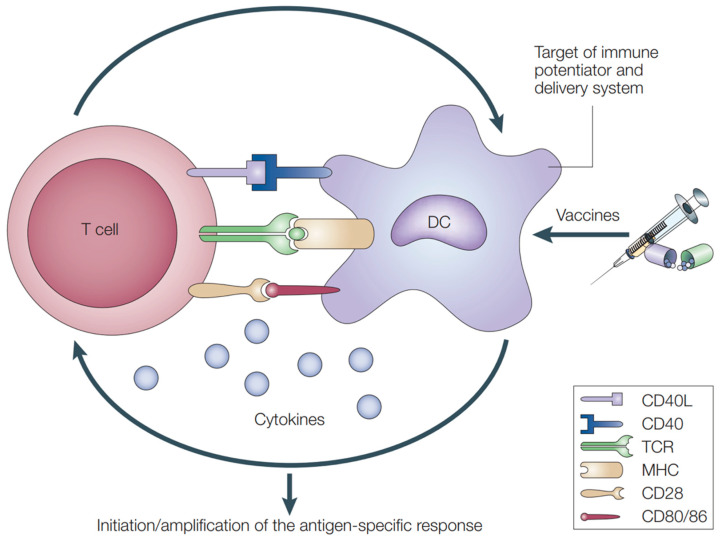
The interactions between APCs and antigen-specific T cells. Adapted with permission from [150]. Copyright 2003, Springer Nature.

From a mechanistic view, an immune response starts with sampling and presentation of antigens by APCs. Evidence has demonstrated that adjuvants can activate APCs, facilitate antigen uptake and cross-presentation between APCs and T-cells, and stimulate the production of immunoregulatory molecules [147,148]. In addition, the importance of adjuvants is underlined when they are exploited to direct the desired types of immune response (e.g., type-1 immunity versus type-2 immunity, CD8^+^ versus CD4^+^ T-cells) and promote the generation of immunological memory [135]. Based on their modes of action, adjuvants can be grouped into two main categories, delivery systems and immune potentiators [141]. Delivery systems act as carriers or depots where antigens and other vaccine components can stay and maintain their stability; in the meantime, they create local proinflammatory responses and recruit APCs. Immune potentiators can activate innate immune cells directly or through PRRs (Figure 3), such as TLRs, nucleotide-binding oligomerization domain (NOD)-like receptors (NLRs), and retinoic acid-inducible gene-I (RIG-I)-like receptors (RLRs) [135]. The interactions between PRRs and pathogen-associated molecular patterns (PAMPs) activate innate immune cells to produce chemokines and cytokines [140]. Once activated, APCs will present antigens to T-cells via MHC and release costimulatory molecules to prime naïve T-cells, bridging the fast-acting innate response with antigen-specific adaptive response (Figure 4). 

### 3.1. Delivery Systems

Particulate delivery systems consisting of synthetic polymers have been widely used in cancer vaccines in recent years. A Pickering emulsion adjuvant system (PPAS) was developed to improve the interactions between antigens and APCs, thus enhancing the efficacy and safety of vaccination [151]. Poly (lactide-co-glycolic acid) (PLGA) nanoparticles were adsorbed to the surface of squalene droplets and served as colloidal stabilizers for the new version of classical microemulsion adjuvant MF59. After subcutaneous injections in C57BL/6 mice, PPAS triggered recruitment of APCs and boosted antigen uptake. The activation of APCs was more efficient than ovalbumin (OVA)-loaded nano/microparticles, OVA-loaded MF59 and OVA alone. Increased quantity of OVA and presenting DCs were found accumulated in the draining lymph nodes in the mice dosed with PPAS compared with those injected with other adjuvant systems or antigen alone. In addition, PPAS-induced OVA-specific immunity protected mice from the challenge of E.G7/OVA lymphoma cells by delaying the tumor growth and maintaining high survival rates compared to solid particles or classical emulsions. As a potential therapeutic adjuvant, PPAS was formulated with MUC1 peptides as a vaccine against B16/MUC1 melanoma. Tumor growth was inhibited in mice vaccinated with MUC1-PPAS and 7/8 mice survived for over 28 days after the first dose of vaccine. Two mice survived to the end of the study (80 days) but those treated with other vaccines could not live for longer than 40 days post-tumor inoculation. These results suggested that PPAS could be a promising adjuvant for cancer immunotherapy. 

**Figure 5:**
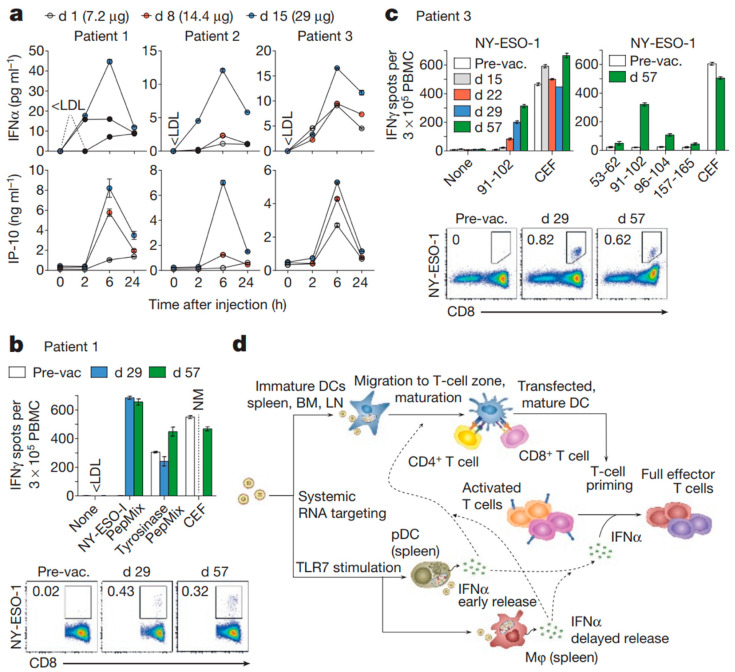
(**a**) Serum cytokines before (0 h) and after injection of intra-patient escalated doses. (**b**,**c**) T-cell responses against NY-ESO-1 and tyrosinase determined by restimulation with overlapping peptide mixtures or NY-ESO-1 epitopes (indicated with the amino acid position) in IFNγ ELISPOT- and NY-ESO-1-specific MHC class I dextramer staining for patients 1 (**b**) and 3 (**c**). CEF, cytomegalovirus, Epstein–Barr and influenza viruses peptide pool; NM, not measured; PepMix, peptide mixture; pre-vac., pre-vaccination. (**d**) Mechanism of action for RNA-LPX. Error bars, mean ± s.e.m. Reprinted with permission from [152]. Copyright 2016, Springer Nature.

Spleen-targeted lipoplexes (LPX) were developed as a cancer vaccine to deliver antigen-encoding RNA to APCs in lymphoid organs via intravenous (IV) administration (Figure 5) [152]. The optimized RNA-LPX was formulated by manipulating the charge ratio between broadly used cationic lipid (DOTMA or DOTAP/DOPE) and RNA, aiming to maintain the selective antigen expression in splenocytes without compromising the colloidal and biological stability of RNA-LPX. This study has identified that macropinocytosis was the major mechanism of DCs uptake of RNA-LPX. A single IV dose of RNA-LPX encoding influenza hemagglutinin (HA) could activate DCs, NK, B, CD4^+^, and CD8^+^ T cells followed by interferon α (IFNα) burst in the blood whereas lipid vehicles could not. Therapeutic effect of RNA-LPX was first evaluated in mice bearing lung metastases derived from colon cancer CT26 expressing gp70, and melanoma B16 expressing OVA, or TRP-1 respectively. Tumors were eradicated by antigen-encoding RNA-LPX in these tumor models by inducing strong IFNα response in lymphoid tissues. LPX loaded with viral oncogene-coding or neoantigen-encoding RNA significantly inhibited tumor growth and generated immune memory in mice bearing TC-1 or CT26 tumors. The safety and tolerability of the RNA-LPX vaccine (Lipo-MERIT) was assessed in patients with advanced melanoma in a phase I/II dose-escalation trial (NCT02410733) [153]. Lipo-MERIT was well-tolerated, and no dose-limiting toxicities were observed in more than 50 patients who received escalating or constant dosing. 

**Figure 6:**
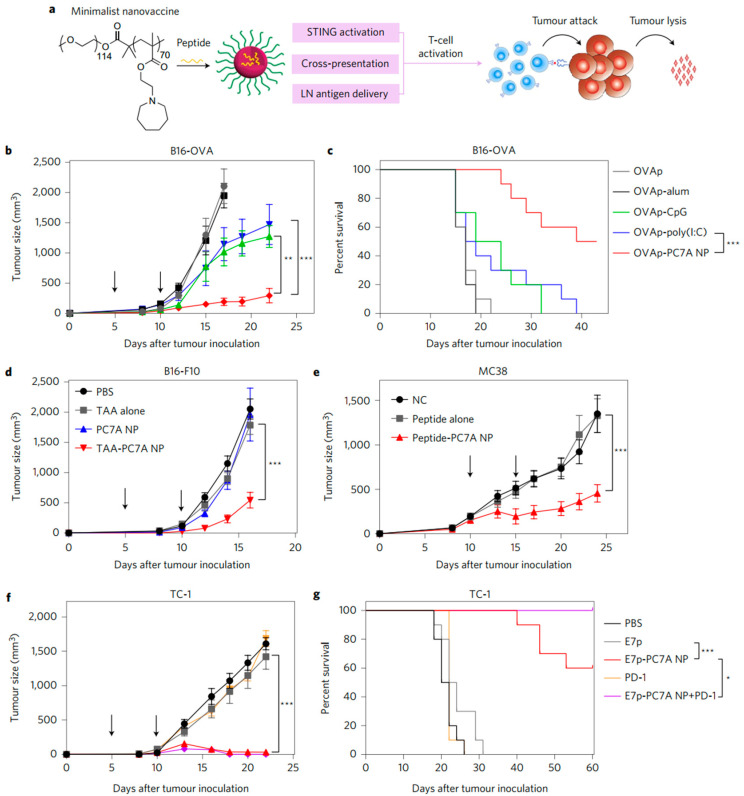
(**a**) Schematic of the minimalist design of the PC7A nanovaccine. (**b**,**c**) C57BL/6 mice (*n* = 10 per group) inoculated with 1.5 × 10^5^ B16-OVA tumor cells were treated with OVA peptide, PC7A nanovaccine, CpG, poly(I:C) and alum plus peptide (0.5 µg). Tumor growth (**b**) and Kaplan–Meier survival curves (**c**) of tumor-bearing mice are shown. (**d**) Tumor growth inhibition study of B16-F10 melanoma. C57BL/6 mice (*n* = 10 per group) inoculated with 1.5 × 10^5^ B16-F10 tumor cells were treated with a cocktail of tumor-associated antigens (Gp100_21–41_, Trp1_214–237_, Trp2_173–196_) in PC7A NPs at specific time points, indicated by arrows. (**e**) Tumor growth inhibition study of MC38 colon cancer in C57BL/6 mice. Mice (*n* = 10 per group) inoculated with 1.0 × 10^6^ MC38 tumor cells were treated with a cocktail of neoantigens (Reps1_P45A_, Adpgk_R304M_, Dpagt1_V213L_) in PC7A NPs, and nanovaccine was administered on days 10 and 15 in established tumors (100–200 mm^3^). (**f**,**g**) In the HPV tumor model, tumor growth inhibition (**f**) and survival data (**g**) in C57BL/6 mice (*n* = 10 per group) were analyzed after tumor inoculation with 1.5 × 10^5^ TC-1 tumor cells. In (**b**,**d**–**f**) data are presented as means ± s.e.m. Statistical significance was calculated by Student’s *t*-test: *** *p* < 0.001, ** *p* < 0.01. Statistical significance for survival analysis in (**c**,**g**) was calculated by the log-rank test: *** *p* < 0.001, * *p* < 0.05. Reprinted with permission from [154]. Copyright 2017, Nature Springer.

In a study of stimulator of interferon genes (STING)-targeted vaccines, Luo et al. synthesized a series of pH-sensitive polymers and found one of them, PC7A—a copolymer containing a tertiary amine with a cyclic side chain, could form ~30-nm particles and activate STING by itself [154]. DCs were found to be the major cell population which captured PC7A NPs, and the activation of type I interferons (IFNs) pathway depended on the specific interactions between PC7A and STING. PC7A was formulated with tumor-associated peptide antigens or neoantigens as vaccines against melanoma B16F10 and colon cancer MC38. Tumor growth was significantly suppressed in the mice treated with PC7A nanovaccines compared to those treated with antigen or blank particles alone. A synergistic antitumor effect was observed when PC7A NPs were administered with anti-PD-1 antibodies in mice bearing TC-1 or B16-OVA tumors. A total of 90% of mice survived tumor-free over 60 days after treatment with PC7A NPs plus anti-PD-1, which was longer than those injected with any single treatment (Figure 6). Immune memory generated by PC7A nanovaccines protected tumor-free mice from the re-challenge of TC-1 cells. These results demonstrated that PC7A vaccine was a simple but potent treatment to boost antitumor immunity. 

In addition to particles made of synthetic polymers, hydrogels/cryogels comprised of biomaterials and their derivatives such as alginate, collagen, and hyaluronic acid [155,156,157,158], have been exploited to control the release of antigens and adjuvants, aiming to improve the efficacy of cancer vaccines [159]. The Mooney lab has developed macroporous cryogels as cancer vaccines which were made of crosslinked methacrylated alginate and loaded with CpG ODN 1826, granulocyte-macrophage colony-stimulating factor (GM-CSF) and irradiated B16F10 cells. These cryogels elicited local DC infiltration and induced potent and durable anti-tumor responses against melanoma. To further improve the injectability, tougher cryogel vaccines were fabricated by incorporating calcium ions as ionic crosslinkers. The tough cryogels could protect 80% of the vaccinated mice for more than 150 days against the challenge of HER2/neu-overexpressing breast cancer [155,156].

Chitosan is a cationic polysaccharide with low toxicity and good biocompatibility [160]. Chitosan hydrogel could work as a depot for antigens to trigger protective CD8^+^ T-cell memory against cancer [161,162]. In a study of chitosan-based nanovaccines, chitosan NPs were functionalized with mannose (Man) for targeted delivery of B16 melanoma cell lysate (TCL) to DCs [163]. Mice vaccinated with Man-chitosan NPs loaded with TCL had an increased population of CD3^+^CD8^+^ T lymphocytes in draining lymph nodes and spleens, and hence could resist the subsequent challenge of B16 tumor cells. When used as a therapeutic vaccine, Man-chitosan NPs could remarkably suppress the proliferation of B16 tumors compared to the untargeted vaccine, blank NPs or TCL alone. These results demonstrated that mannose-decorated chitosan NPs were promising vehicles for targeted delivery of antigens for cancer immunotherapy. In addition, a chitosan derivative, N-dihydro-galacto-chitosan (GC), was utilized as an immunoadjuvant in laser immunotherapy in which a laser fiber was inserted into an accessible tumor to cause immunogenic cell death and release antigens by heat [164,165]. GC solution was injected into the heat-treated tumor mass to enhance the immune responses. The combination of photothermal treatment and GC adjuvant have shown promise in the treatment of both primary and metastatic tumors in some breast cancer patients [166]. 

### 3.2. Immune Potentiators 

In contrast to empirically derived adjuvants such as alum, MF59 or saponins, recent studies have focused on the well-defined PRRs and most immune potentiators are agonists for TLRs, NLRs, RLRs, and cGAS-STING [140]. TLRs are expressed in a variety of immune cells including mast cells [167], DCs [168], macrophages [169], NK cells [170], B-cells [171], T cells [172], and some non-immune cells such as epithelial cells [173], endothelial cells [174], and fibroblasts [175]. Humans possess TLR1 to 10, which are expressed on different cellular localizations. TLR1, 2, 4, 5, and 6 are situated on the plasma membrane, sensing microbial and fungal cell walls [140]. TLR3, 7, 8, and 9 are expressed in endosomal compartments, which can detect bacterial and viral nucleic acids and stimulate the production of Type I IFNs, initiating innate immunity against cancers [176,177]. TLR10 is a unique member of TLR family which was found capable of suppressing TLR signaling and immune responses. The biological function and ligand of TLR10 still need further investigation [178]. STING is a cytosolic double-stranded DNA sensor located in the endoplasmic reticulum, which is mainly stimulated by cyclic dinucleotides (CDN) generated by cyclic GMP-AMP synthase (cGAS) [177]. STING has been well-characterized in APCs, and recent studies have reported its roles in T lymphocytes, endothelial cells and fibroblasts present in the tumor microenvironment (TME) [179,180,181,182]. cGAS-STING senses not only foreign DNA from bacteria or virus, but also self-DNA released by damaged or dying cells, including cancer cells [183]. RLRs, such as melanoma differentiation-associated 5 (MDA5), RIG-I, and laboratory of genetics and physiology 2 (LGP2), are cytoplasmic sensors for double-stranded RNA (e.g., poly I:C) which are expressed at a low level in most tissues. The RLR family plays a critical role in amplifying immune responses because its expression can be upregulated by type I IFN [184,185]. NLRs are a large family of cytoplasmic receptors detecting a variety of PAMPs and danger-associated molecular patterns (DAMPs) [186,187,188]. Many efforts have been taken to explore the potential of NOD2 ligands (e.g., muramyl dipeptide and its derivatives) as adjuvants for human use due to their abilities to induce type I IFN production and cellular immunity [189,190]. Given the cytosolic locations of the aforementioned PRRs, targeted delivery of agonists may facilitate the activation of correlative signaling pathways, leading to efficient immune responses. 

### 3.3. Intracellular Delivery of Immune Adjuvants

#### 3.3.1. TLR3

As a ligand to TLR3, Poly(I:C) has been widely utilized as a vaccine adjuvant or combined with antibodies for immunotherapies against various cancers [191,192,193,194]. However, poly(I:C), a synthetic mimic of viral double-strand RNA (dsRNA), suffers poor penetration through cell membrane and could be easily degraded by serum nucleases [195]. To facilitate intracellular trafficking of poly(I:C), cationic materials have been used to deliver negatively charged dsRNA. Han et al. developed chitosan-based vaccines to deliver poly(I:C) and OVA or E7 peptides to stimulate antigen-specific DC maturation [196]. These chitosan NPs showed efficient intracellular uptake and specific release of poly(I:C) and antigens in acidic cellular environment. After intraperitoneal injection of chitosan NPs, an increased number of activated DCs and antigen-specific CD8^+^ T-cells were identified in the peritoneal lymph nodes and spleen respectively. Antitumor efficacy was evaluated in mice bearing EG.7-OVA or TC-1 tumors. Tumor growth in both models was significantly suppressed and the survival of vaccinated mice was prolonged compared to those treated with soluble antigens or blank NPs. Another dsRNA adjuvant Riboxxim (RGIC^®^, Riboxx Pharmaceuticals, Dresden, Germany) has superior stability and lower toxicity than poly(I:C) [197,198]. It can activate murine and human DC and induce TLR3/RIG-I mediated antitumor immunity and demonstrated great potential in cancer immunotherapy [199]. 

#### 3.3.2. TLR7/8

**Figure 7:**
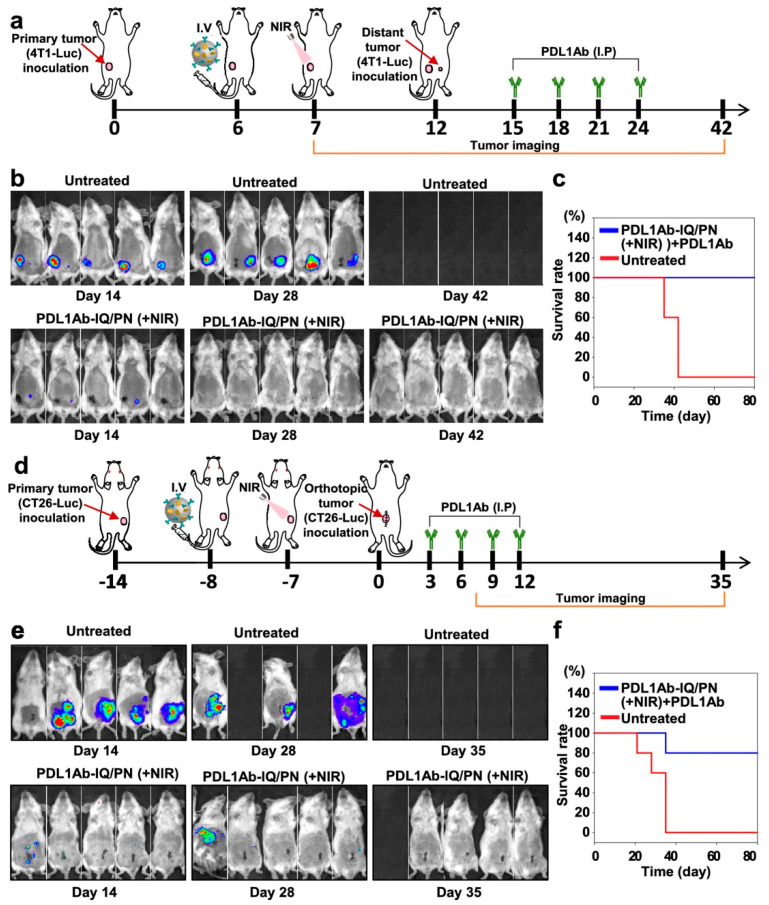
In vivo antitumor efficacy of PDL1Ab-PNs against orthotopic tumors. (**a**) Outline of 4T1 orthotopic breast tumor inoculation and dosing regimen. The 4T1 orthotopic breast tumor-bearing mice were intravenously injected with PDL1Ab-IQ/PNs on day 6 after inoculation and irradiated with an 808-nm laser on day 7. Twelve days after the first tumor inoculation, 4T1 cells were inoculated at a site distant from the primary tumor. In the untreated group, primary tumors were surgically removed on day 18 for observation of distant orthotopic tumor growth. PDL1Ab (100 μg) were administered on days 15, 18, 21, and 24. (**b**) The formation of orthotopic tumors was observed by luminescence imaging. Black panels in images correspond to mice that died. (**c**) Survival rates of mice monitored for 80 days. (**d**) Illustration of CT26-Luc orthotopic colorectal tumor inoculation and dosing regimen. Mice were intravenously injected with PDL1Ab-IQ/PNs 7 days after inoculation with CT26 primary tumor and irradiated with an 808-nm laser the next day. Fourteen days after the first tumor inoculation, an orthotopic colorectal tumor was produced by inoculating the cecum with luciferase-expressing CT26-Luc cells. PDL1Ab (100 μg) were administered on days 3, 6, 9, and 12 after inoculation with the orthotopic distant tumor. (**e**) The growth of orthotopic tumors was observed by luminescence imaging until day 35 after orthotopic tumor inoculation. (**f**) Survival rates of mice monitored for 80 days. Reprinted with permission from [204]. Copyright 2022 American Chemical Society.

Imidazoquinolines are synthetic small molecule immune potentiators capable of activating and recruiting DCs [200,201]. Imiquimod can induce the production of cytokines including interferon-α, tumor necrosis factor-α, and interleukin-1 via TLR7-MyD88-dependent pathway [202,203]. In a combination therapy using photothermal nanoparticles and immune checkpoint inhibitors, Imiquimod (R837) was encapsulated in polydopamine nanoparticles which were further modified with anti-PDL1 antibodies on the surface (PDL1Ab-IQ/PNs) via Michael addition [204]. In this study, polydopaminenot only served as a photo-responsive material to near-infrared irradiation, but also as a vehicle for hydrophobic drug R837 and PDL1 antibody. After intravenous administration, these antibody-bound nanoparticles showed higher tumor accumulation than unmodified particles due to overexpressed PDL1 on CT26 or 4T1 cancer cells. Local near-infrared (NIR) irradiation was subsequently given to tumors so that tumor antigens were liberated by the photothermal effect generated by polydopamine. R837-loaded polydopamine nanoparticles and tumor antigens were taken up and digested by the same DCs which suggested that co-delivery of tumor antigens and adjuvants to APCs could induce more durable and stronger immune responses than separate treatment. With the in-situ assembly of adjuvant, antigen, and immune checkpoint inhibitor, 4T1 or CT26 tumor was eradicated, and a secondary tumor challenge was prevented at a distant site, achieving a survival for up to 80 days (Figure 7). Resiquimod (R848) is a dual agonist of TLR 7 and 8 which has been utilized together with poly-ICLC to activate APCs and induce immunity in cancer patients in phase I clinical trials [205,206]. In addition, R848 can lower the accumulation of regulatory T-cells and myeloid-derived suppressor cells present in the tumor microenvironment to benefit immune checkpoint blockade therapy [207,208]. However, the toxicity of R848 constrained Its clinical application [209,210]. Various strategies, including prodrug, hydrogel depot, and NP encapsulation [211,212], have been developed to deliver R848 safely. Lu et al. developed a hydrogel depot to deliver a R848-tocopherol prodrug intratumorally in a canine model with mast cell tumors and yielded a complete remission and three partial remissions without significant immune-related adverse effects [213].

#### 3.3.3. TLR9

CpG-oligodeoxynucleotide (ODN) is a widely used adjuvant containing unmethylated CpG motifs which can be recognized by TLR9, promoting antigen-specific adaptive immune responses against infectious diseases and cancer [214,215]. Three classes of ODNs have been developed and the wholly phosphorothioate C-Class ODN (e.g., 2395, 2429), a combination of A- and B-Class, has shown its strong potency as a Th1-promoting adjuvant [216]. Several strategies have been developed to deliver CpG. As a water-soluble single-stranded ODN, CpG can be delivered in alginate-based cryogels or scaffolds [156,217], or electrostatically bound to polyethylenimine (PEI) [218]. There are more examples of loading CpG via covalent conjugation to lipid materials [219,220,221,222] or polyethylene glycol hydrogel via disulfide bonds [223].

**Figure 8:**
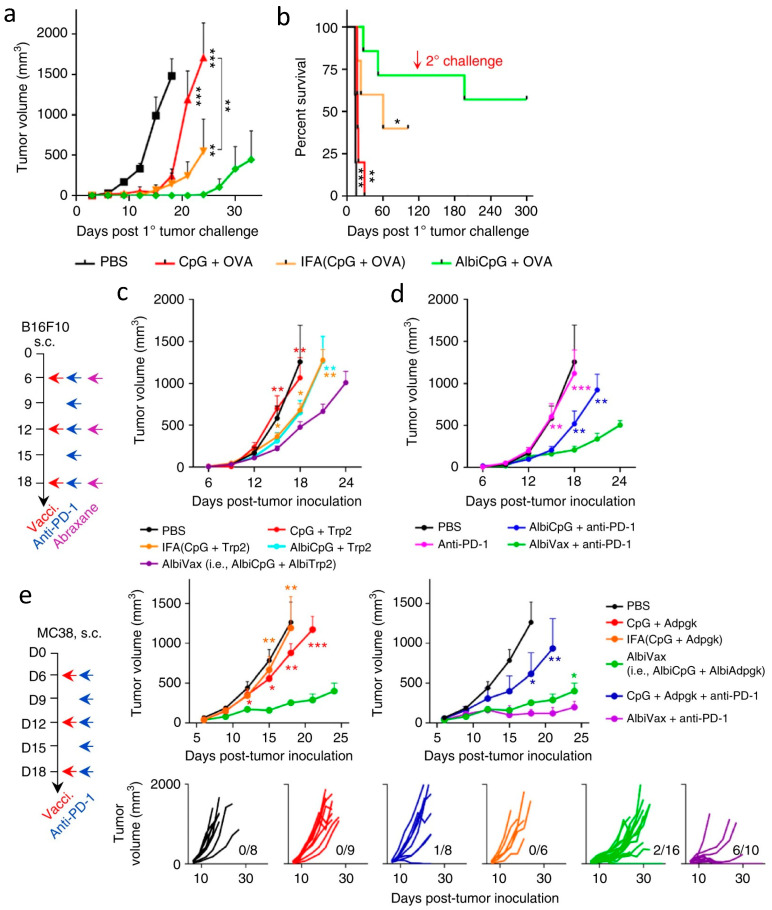
(**a**,**b**) Tumor growth curves and mouse survival after s.c. challenging vaccinated mice with EG7.OVA cells. The 1° challenge: 3 × 10^5^ cells on the right shoulder on day 71 post priming vaccination; the 2° challenge: 1 × 10^6^ cells on the right flank on day 211. (**c**,**d**) B16F10 tumor growth after treatment with AlbiVax, double combination of albumin/AlbiVax nanocomplexes and anti-PD−1. C57BL/6 mice were s.c. inoculated with 3 × 10^5^ B16F10 cells, treated with AlbiVax (2 nmol CpG equivalents + 20 µg AlbiTrp2) (day 6, day 12, and day 18), anti-PD-1 every 3 days from day 6 for five times (200 µg). (**e**) MC38 tumor growth after treatment with AlbiVax alone or in combination with anti-PD-1. C57BL/6 mice were s.c. inoculated with 3 × 10^5^ MC38 cells, treated with AlbiVax (2 nmol AlbiCpG + 20 µg AlbiAdpgk) on day 6, day 12, and day 18, and with anti-PD-1 (200 µg) every 3 days from day 6 for six times. *** *p* < 0.001, ** *p* < 0.01, * *p* < 0.05, by one-way ANOVA with Bonferroni post-test. Adapted from [225] with Creative Commons CC BY license.

Cholesterol-modified CpG was delivered together with neo-epitopes in disc-shaped nanocomplexes formed by synthetic high-density lipoprotein (nanodisc) [224]. When combined with anti-PD-1 antibodies, the nanodisc vaccine generated strong cytotoxic T-lymphocyte responses in mice bearing MC38 tumors. About 88% of mice had complete tumor regression compared to only 25% of those treated with soluble vaccine and anti-PD-1. Furthermore, multiple peptide antigens of melanoma were loaded in nanodiscs which were administered with anti-CTLA-4 and anti-PD-1 simultaneously. B16F10 tumors were eradicated in 9/10 of mice, whereas soluble vaccine and dual inhibitors led to complete regression in only 3/8 of mice. In an effort to exploit endogenous albumin as a vaccine carrier, an in-situ assembly vaccine (AlbiVax) was constructed by conjugating thiol-modified CpG or antigen peptide to maleimide-functionalized Evans blue dye (EB), which can strongly bind to human serum albumin after vaccination [225]. This strategy exploits clinically safe EB and endogenous albumin to achieve lymph node accumulation and intracellular delivery of peptide antigens and adjuvants to APCs. AlbiVax showed significantly higher lymph nodes accumulation and CD8^+^ T-cells activation than incomplete Freund’s adjuvant. When equipped with specific peptide antigens, these vaccines markedly inhibited the tumor growth of MC38, EG7.OVA, and B16F10. Complete regressions were observed in 6 out of 10 mice bearing MC38 tumor after receiving the combined therapy of AlbiVax and anti-PD-1 (Figure 8). These results demonstrated that AlbiVax was a simple and robust nanovaccine for cancer immunotherapy. 

#### 3.3.4. cGAS-STING

**Figure 9:**
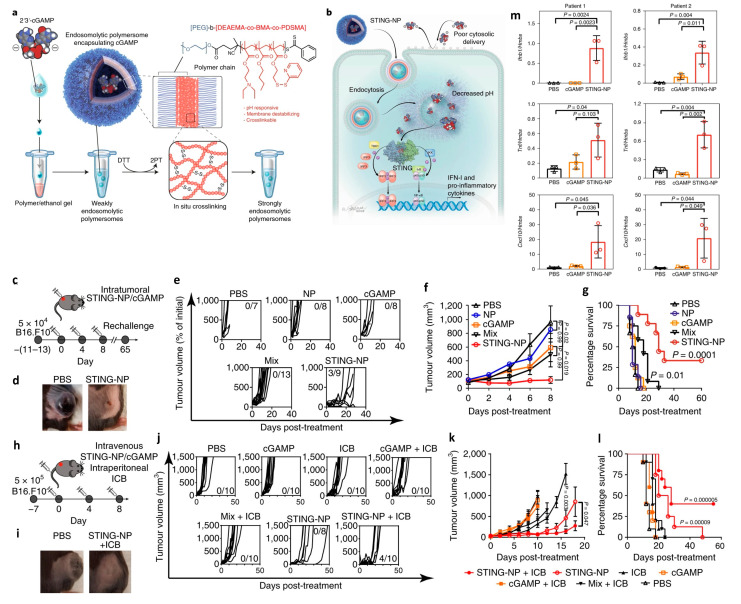
(**a**) Schematic of the STING-NP structure and strategy for enhancing intracellular delivery of 2′3′-cGAMP. cGAMP is encapsulated in endosomolytic polymersomes assembled from pH-responsive diblock copolymers. After polymersome self-assembly and cGAMP loading, polymer chains are crosslinked in situ via partial reduction of pyridyl disulfide groups with DTT, resulting in the formation of disulfide crosslinks. 2PT, 2-pyridinethione. (**b**) STING-NPs enhance intracellular uptake of cGAMP and, in response to decreased pH within endosomal compartments, disassemble and promote endosomal escape of cGAMP to the cytosol. IKK, IκB kinase; IκB, inhibitor of κB; IRF3, IFN regulatory factor 3; TBK1, TANK-binding kinase 1. (**c**) Intratumoral administration and tumor rechallenge scheme for mice with a single established B16.F10 tumor. Mice with 100 mm^3^ subcutaneous tumors were administered STING-NPs, free cGAMP, empty nanoparticles (NPs), a physical mixture of empty NPs and cGAMP (mix), or PBS intratumorally three times, 4 d apart. (**d**) Photographs of tumors 8 d after treatment. The experiment was conducted three times independently with similar results. (**e**) Spider plots of individual tumor growth curves, with the numbers of complete responses denoted. The experiment was conducted three times independently with similar results. (**f**) Mean tumor volume from three independent experiments (for PBS, NP, cGAMP, mix and STING-NP; *n* = 7, 8, 8, 13 and 9 biologically independent samples, respectively; Kruskal–Wallis test with Dunn’s multiple comparisons test). (**g**) Kaplan–Meier survival curves of mice treated with the indicated formulation using a 1500 mm^3^ tumor volume as the endpoint criteria (for PBS, NP, cGAMP, mix and STING-NP, *n* = 7, 8, 8, 13 and 9 biologically independent samples, respectively; two-tailed Mantel–Cox test). (**h**) Treatment scheme for mice treated intravenously with cGAMP formulations and intraperitoneally with ICB three times, 4 d apart. (**i**) Representative images of tumors 8 d after initiation of treatment. The experiment was conducted three times independently with similar results. (**j**) Spider plots of individual tumor growth curves of intravenously treated mice. (**k**) Average tumor volume (two-tailed Mann–Whitney *U*-test; *p* = 0.003 denotes the significance of STING-NP relative to ICB). (**l**) Kaplan–Meier survival analysis (two-tailed Mantel–Cox test). In (**j**–**l**) for PBS, cGAMP, ICB, cGAMP + ICB, mix + ICB, STING-NP and STING-NP + ICB, *n* = 10, 10, 10, 10, 10, 8 and 10 biologically independent samples, respectively. All statistical data are represented as means ± s.e.m. (**m**) Surgically resected melanoma metastases were divided into nine sections (three per treatment; one-way ANOVA with Tukey test), randomized, injected intratumorally with STING-NPs or cGAMP at 150 nM and cultured for 24 h. The graphs show the results of qPCR analysis of *Ifnb1*, *Tnf*, and *Cxcl10* gene expression in tissues freshly isolated from two different melanoma patients after the indicated treatment. All statistical data are presented as means ± s.d. Adapted with permission from [227]. Copyright 2019, Springer Nature.

Three types of STING agonists have been developed and evaluated in various immunotherapeutic studies so far. CDNs such as cyclic guanosine monophosphate-adenosine monophosphate (cGAMP) and bis-(3′-5′)-cyclic dimeric guanosine monophosphate (c-di-GMP), are potent adjuvants on the induction of antigen-specific CD8^+^ T cells [226]. Due to their negative charge and high aqueous solubility, these molecules are subjected to low efficient transmembrane transport and inability of activating cytoplasmic STING [227]. Delivery platforms such as nanoparticle vehicles, have been developed to enhance the bioavailability and therapeutic effect of CDNs. C-di-GMP could be encapsulated as a vaccine adjuvant in PEGylated liposomes to facilitate the transport into draining lymph nodes and reduce the risk of systemic inflammation after subcutaneous injection of free c-di-GMP [228]. To enhance APC uptake and activation, polymersomes were constructed with pH-responsive polymers to facilitate cytosolic delivery and endosomal escape of cGAMP [227]. After intratumoral (IT) injection, these STING-activating polymersomes (STING-NPs) have increased the gene expression levels of inflammatory cytokines (interferon-β1, CXCL9 and CXCL10) in harvested tumor cells and shown remarkable uptake by NK cells, DCs and macrophages in TME and draining lymph nodes. Compared to free cGAMP, an increased number of infiltrating T cells were localized in tumors treated with STING-NP, rendering an immunogenic TME. In the therapeutic studies, IT administered STING-NPs significantly suppressed the growth of B16F10 melanoma and prolonged the median survival to 29 days, compared to 12 days in mice treated with free cGAMP (Figure 9). One-third of mice treated with STING-NPs were tumor-free up to 65 days and nearly 70% of them resisted a second tumor challenge for 5 months. When combined with anti-PD-1 and anti-CTLA-4, STING-NPs remarkably inhibited the growth of treated tumor and non-treated distal tumor. STING-NPs exhibited similar antitumor effect when injected systemically yet caused body weight decrease in mice without any other noticeable indications. In addition, human metastatic melanoma specimens from two patients showed elevated expression of several cytokines after treatment with STING-NPs, demonstrating the translational promise of STING-NPs. 

Dimethyloxoxanthenyl acetic acid (DMXAA) was developed as a small molecule chemotherapeutic agent which could disrupt tumor blood supply in several mouse models [229]. Further studies have showed that DMXAA, as a STING agonist, could induce the activation of NK cells and tumor-associated macrophages, leading to necrosis in tumors [230]. However, accumulating evidence indicated that DMXAA was a mouse-specific STING activator due to the difference between human STING (hSTING) and mouse STING at the cyclic-dinucleotide-binding site [230,231,232]. Therefore, efforts have been taken to modify the structure of DMXAA for a better binding to hSTING [233]. A new non-nucleotide STING agonist has been synthesized by linking two molecules of amidobenzimidazole (ABZI), eliciting comparable binding affinity with hSTING but higher potency in STING activation relative to cGAMP [234]. Intravenous administration of di-ABZI resulted in significant and durable tumor regression in CT-26 tumor-bearing mice whereas cGAMP only showed modest efficacy. The safety profile and delivery strategy of di-ABZI still need further investigation. 

#### 3.3.5. Other PRR Agonists

Accumulating studies have demonstrated that the activation of RLRs and NLRs could be facilitated by particulate delivery of adjuvants as well. A RIG-I agonist, 5′pppdsRNA, was co-encapsulated with antigen peptides in lipid calcium phosphate nanoparticles and used as a nanovaccine against colorectal cancer and liver metastasis [235]. When treated with the dsRNA vaccine, tumor tissue recruited an increased CD8^+^ T-cell population without the accompaniment of more T-reg cells and MDSC. The dsRNA vaccine significantly suppressed the growth of primary CT26 tumor, and meanwhile reduced the metastatic lesions on the liver. Another study demonstrated efficient uptake of encapsulated NOD ligands by DCs and subsequent upregulation of co-stimulatory surface markers, cytokine secretion, and enhanced antigen-specific T-cell responses [236,237].

### 3.4. Combined Delivery of PRR Agonists

The recognition of pathogens by the innate immune system usually requires the orchestration of multiple PRRs due to the sophisticated nature of pathogens [140]. The collaboration between PRRs can effectively induce immune responses to invading antigens or endogenous damage-associated molecules. Several studies have demonstrated the synergistic effects within the TLR family or between TLRs and other PRRs (Table 3) when their agonists were delivered simultaneously in micro- or nano-scale particles. The synergy within the TLR family has been summarized in the literature [238]. 

However, combining PRR agonists does not always lead to synergistic effects on the immune responses. For example, in vitro activation of TLR9 and TLR7 could impair the maturation of DCs collected from mouse and human DC in the context of DC vaccine which might be inconsistent with the results in some of the dual adjuvants studies in Table Y [247]. The release of IL-10 resulting from TLR2 stimulation could inhibit the induction of IFN-γ-inducible protein 10, IFN-γ, and IL-12p35 mediated by TLR3 or 4 agonists [248]. The activation of murine RLRs could compromise T helper type 1 and 17 cell responses induced by TLR signaling [249]. The mechanisms by which PRR pathways crosstalk has not been thoroughly clarified yet. The influential factors of PRR interactions may include the association between two or multiple signaling pathways and positive or negative effects of cross-talking on the cytokine productions and feedback loops after concomitant activation of different PRRs [149,238,250].

## 4. Future Perspective

A better understanding of tumors’ microenvironment and immune evasion mechanisms is fundamental to the development of cancer vaccines and other immunotherapies [251]. Personalized vaccines could benefit from the breakthrough advances and complementation between different antigen discovery and prediction platforms such as SEREX, Proteomex, autoantibody-mediated identification of antigens and other bioinformatics techniques. In addition, the mode of antigen/adjuvant delivery is very critical for the success of cancer vaccines. Particle-based vaccine delivery systems have demonstrated their efficacy in the fight of COVID, and their preliminary success will be further scrutinized in the context of cancer therapies. Although vaccines held great promise, cancer patients would obtain better therapeutic outcomes if they could receive cancer vaccines in combination with other immunomodulation (e.g., immune checkpoint inhibitors, agonistic antibodies, TGF-β inhibitors, etc.) and/or conventional cancer therapies such as radiotherapy and chemotherapy in a well-tailored regimen.

## Figures and Tables

**Table 1:** Tumor antigens arising from mutations.

Antigen	MHC-I	MHC-II
α-actinin	HLA-A2 [54]	
ARTC1		HLA-DR1 [55]
B-RAF		HLA-DR4 [56]
β-catenin	HLA-A24 [57]	
BCR-ABL	HLA-A2 [58] HLA-A3 [59] HLA-A11 [60] HLA-B8 [58]	HLA-DR4 [61] HLA-DR9 [62] HLA-DR11 [63] HLA-DR15 [64] HLA-DR1 [65]
Caspase-5	HLA-A2 [66]	
Caspase-8	HLA-B35 [67]	
CDC27		HLA-DR4 [68]
CDK-4	HLA-A2 [69]	
CDK-12	HLA-A11 [70]	
CDK2NA	HLA-A11 [71]	
CLPP	HLA-A2 [72]	
COA-1		HLA-DR4 [73] HLA-DR13 [73]
CSNK1A1	HLA-A2 [70]	
dek-can		HLA-DR53 [74]
EFTUD2	HLA-A3 [75]	
ELF2M	HLA-A68 [76]	
ETV6-AML1	HLA-A2 [77]	
FLT3-ITD	HLA-A1 [78]	
fibronectin		HLA-DR15 [79]
FNDC3B	HLA-A2 [80]	
GAS7	HLA-A2 [70]	
GPNMB	HLA-A3 [75]	
HAUS3	HLA-A2 [70]	
HSDL1	HLA-Cw14 [81]	
HSP70-2	HLA-A2 [82]	
KIA A0205	HLA-B44 [83]	
K-ras	HLA-B35 [84] HLA-Cw8 [85]	
LDLR-FUT		HLA-DR1 [86]
MART-2	HLA-A1 [87]	
MATN	HLA-A11 [70]	
ME1	HLA-A2 [88]	
MUM-1	HLA-B44 [89]	
MUM-2	HLA-B44 [90] HLA-Cw6 [90]	
MUM-3	HLA-A68 [91]	
Myosin-m	HLA-A3 [92]	
N-ras	HLA-A1 [93]	
neo-PAP		HLA-DR7 [94]
NFYC	HLA-B52 [95]	
OGT	HLA-A2 [96]	
OS-9	HLA-B44 [97]	
p14ARF	HLA-A11 [71]	
p16INK	HLA-A11 [71]	
pml-RARalpha fusion protein		HLA-DR11 [98]
PPP1R3B	HLA-A1 [70]	
PRDX5	HLA-A2 [99]	
PTPRK		HLA-DR10 [100]
RBAF600	HLA-B7 [75]	
SIRT2	HLA-A3 [75]	
SNDRP1	HLA-B38	
SYT-SSX1 or -SSX2 fusion protein	HLA-B7 [101]	
TGFBRII		HLA-DR3 [102]
TP53	HLA-A2 [103]	
TPI		HLA-DR1 [104]
Annexin II		HLA-DR4 [105]

**Table 2:** Clinically tested human vaccine adjuvants. Adapted with permission from [135,140,141]. Copyright 2017, Elsevier.

Adjuvants	Major Immunostimulatory Component(s)	Innate Receptors or Pathway Activated	Status
Alum	Aluminum salts	NLRP3/NALP3 inflammasome complex [142,143,144]	Licensed (Diphtheria, tetanus, pneumococcus, MenC, MenB, and many others)
MF59, AS03	Squalene-in-water emulsions	MyD88	Licensed (influenza)
AS04	MPL adsorbed to alum	TLR4	Licensed (HBV, HPV)
Virosomes	Viral glycoproteins and membrane lipids	Receptor-mediated endocytosis by APCs [140,145,146]	Licensed (influenza)
RC529	Synthetic TLR4 ligand adsorbed to alum	TLR4	Licensed (HBV)
Flagellin	Flagellin from *S. typhimurium*	TLR5	Phase II (influenza, NCT00921947)
Monophosphoryl lipid A (MPL) and formulations (AS01, AS02)	MPL, Saponin (QS21)	TLR4	Phase II (TB, NCT00146744; cancer, NCT00290355) Phase III (malaria, NCT00866619; herpes zoster, NCT01165177)
Imiquimod and Resiquimod	Imidazoquinoline derivatives	TLR7, TLR8 or both	Licensed (skin cancer)
CpG ODN and formulations (IC31)	Synthetic phophorothioate-linked DNA oligonucleotides with optimized CpG motifs	TLR9	Phase I/IIa (malaria, NCT00984763; influenza, NCT03945825) Phase II/III (HBV, NCT01005407; breast cancer, NCT00043394; anthrax, NCT03877926; TB, NCT02075203)
AS15	Liposomes, MPL, CpG, saponin (QS21)	TLR4 and 9	Phase III (melanoma, NCT00796445)
GLA-SE	Oil-in-water emulsion with synthetic TLR4 ligand (GLA)	TLR4	Phase II (influenza, NCT01991561)
Alum/TLR7	Small-molecule synthetic TLR7 ligand adsorbed to alum	TLR7	Phase I (MenC, NCT02639351)
RNAdjuvant^®^ (CV8102)	Single-stranded, non-coding U-rich RNA complexed with crosslinked cationic peptide	TLR7/8/RIG-I	Phase I/II (skin cancer, NCT03291002; hepatocellular carcinoma, NCT03203005)

HBV: hepatitis B virus, HPV: human papillomavirus, MenB and MenC: meningitis B and meningococcal C, TB: tuberculosis.

**Table 3:** Vaccines with combined adjuvants.

Adjuvant Combination	Delivery System	Outcome	Ref.
MPLA (TLR4) Imiquimod (TLR7)	Mannose-functionalized lipid hybrid polymersomes formed with DOTAP, PCL-PEG-PCL and Mannose-PEG-DSPE	Greater prophylactic effect against E.G7-OVA tumor challenge than free OVA plus adjuvants. Greater therapeutic effect when used together with anti-PD1 against E.G7-OVA tumor, comparing to untargeted/targeted polymersomes alone or free OVA plus adjuvants.	[239,240]
CpG (TLR9) with MPLA (TLR4) or R848 (TLR7/8)	PLGA/PEI nanoparticles	After in vivo administration, either combination could induce a significantly higher IFN-γ secretion comparing to single adjuvant or alum; MPLA and CpG could induce more IL-4 secretion than R848 and CpG. As for antibody responses, R848 and CpG could induce a high titer of IgG2a while MPLA and CpG could induce the highest titer of IgG1, which were comparable to alum.	[241]
MPLA (TLR4) CpG (TLR9)	Mannosylated PLGA/PLA nanoparticles	Superior prophylactic effect against melanoma challenge when combined with anti-PD1 and anti-OX40, comparing to nanoparticles alone or other free formulations. Ibrutinib, a MDSC inhibitor, was needed to inhibit melanoma growth at the therapeutic regimen.	[242]
Poly(I:C) (TLR3) CpG (TLR9)	Liposomes prepared with phospholipid and cholesterol	Dual TLR-adjuvant vaccine protected 6 out of 8 mice from tumor challenge of OVA expressing E.G7 in 8 months after primary and booster injections.	[243]
β-glucan (TLR2) CpG (TLR9)	Aminated glucan nanoparticles (AG NP)	CpG-AG NP elevated antigen-specific antibody titers in serum (IgG1 and IgG2a) and the production of IL-4 and IFN-γ in immunized mice, which were comparable to Freund’s adjuvant without causing any significant toxicity.	[244]
R848 (TLR7/8) cGAMP (STING)	Acetalated dextran microparticles (Ace-DEX MP)	Dual-adjuvant Ace-DEX MP induced a higher production of IL-2 and IFN-γ than Alum in immunized mice. This combination also induced elevated overall antibody titers with a near even ratio of IgG2c/IgG1, achieving a balanced Th1/Th2 humoral responses.	[245]
R848 (TLR7/8) CpG (TLR9)	PEG-PLA micelles	Bi-adjuvant nanovaccine loaded with neoantigen Adpgk induced potent antitumor immunity against MC38 colorectal cancer without causing acute systemic toxicity.	[246]

## Data Availability

Not applicable.

## Supplementary Materials

The following supporting information can be downloaded at: https://www.mdpi.com/article/10.3390/pharmaceutics14071448/s1, Table S1: Differentiation tumor antigens; Table S2. Overexpressed tumor antigens; Table S3. Tumor-specific antigens. References [7,15,75,105,252,253,254,255,256,257,258,259,260,261,262,263,264,265,266,267,268,269,270,271,272,273,274,275,276,277,278,279,280,281,282,283,284,285,286,287,288,289,290,291,292,293,294,295,296,297,298,299,300,301,302,303,304,305,306,307,308,309,310,311,312,313,314,315,316,317,318,319,320,321,322,323,324,325,326,327,328,329,330,331,332,333,334,335,336,337,338,339,340,341,342,343,344,345,346,347,348,349,350,351,352,353,354,355,356,357,358,359,360,361,362,363,364,365,366,367,368,369,370,371,372,373,374,375,376,377,378,379,380,381,382,383,384,385,386,387,388,389,390,391,392,393,394,395,396,397,398,399,400,401,402,403,404,405,406,407,408,409,410,411,412,413,414,415,416,417,418,419,420,421,422,423,424,425,426,427,428,429,430,431,432,433,434,435,436,437,438,439,440,441,442,443,444,445,446,447,448,449,450,451,452,453,454,455,456,457,458,459,460,461,462,463,464,465,466,467,468,469,470,471,472,473,474,475,476,477,478,479,480,481,482,483,484,485,486,487,488,489,490,491,492,493,494,495] are cited in the supplementary materials.
