# Supplementary material for: Therapeutic Cancer Vaccines—Antigen Discovery and Adjuvant Delivery Platforms
Source: Pharmaceutics. 2022 Jul 11;14(7):1448. doi: 10.3390/pharmaceutics14071448 (PMC9325128; doi:10.3390/pharmaceutics14071448)
Supplement: Supplementary file 1 [file pharmaceutics-14-01448-s001.zip › pharmaceutics-1758382-supplementary.pdf]

Tables S1–S3 were compiled from work done at the de Duve institute and does not reflect the entirety of the peptides that can induce an adaptive immune response, but rather which MHCs are activated. For a more complete list of the peptides that induce an adaptive immune response, please visit the de Duve institutes website https://caped.icp.ucl.ac.be/Peptide/list.

Table S1. Differentiation tumor antigens.

Antigen	MHC-I	MHC-II
AIM-1	HLA-A2[1]	
CEA	HLA-A2[2, 3]	HLA-DR3[8]
	HLA-A3[4, 5]	HLA-DR4[9]
	HLA-A24[6]	HLA-DR7[8]
	HLA-B27[7]	HLA-DR9[10]
		HLA-DR11[11]
		HLA-DR13[11]
		HLA-DR14[11]
Gp-100	HLA-A2[12, 13]	HLA-DQ6[20]
	HLA-A3[14]	HLA-DR1[21]
	HLA-A11[14]	HLA-DR4[22]
	HLA-A24[15]	HLA-DR7[23]
	HLA-A68[16]	HLA-DR53[20]
	HLA-B7[17]	
	HLA-B35[18]	
	HLA-Cw8[19]	
Mammaglobin-A	HLA-A3[24]	
MART-1	HLA-A2[25, 26]	HLA-DR1[29]
	HLA-B35[18]	HLA-DR3[29]
	HLA-B45[27]	HLA-DR4[30]
	HLA-Cw7[28]	HLA-DR11[31]

Antigen	MHC-I	MHC-II
		HLA-DR52[31]
MC1R	HLA-A2[32]	
NY-BR-1	HLA-A2[33]	
OA1	HLA-A24[34]	
PAP	HLA-A2[35]	
PSA	HLA-A2[36]	HLA-DR4[37]
P. polypeptide	HLA-A2[38]	
RAB38 / NY-MEL-1	HLA-A2[39]	
SOX-10	HLA-A2[40]	
TRP-1(gp75)	HLA-A31[41]	HLA-DR4[42]
		HLA-DR15[43]
		HLA-DR17[44]
TRP-2	HLA-A2[45]	HLA-DR3[47]
	HLA-A31[46]	HLA-DR15[43]
	HLA-A33[46]	
	HLA-Cw8[19]	
Tyrosinase	HLA-A1[48]	HLA-DR4[54]
	HLA-A2[49, 50]	HLA-DR15[55]
	HLA-A24[51]	
	HLA-A26[17]	
	HLA-B35[52]	
	HLA-B38[17]	
	HLA-B44[53]	

Table S2. Overexpressed tumor antigens.

Antigen	MHC-I	MHC-II
Adipophilin	HLA-A2[56]	
AIM-2	HLA-A1[57]	
ALDH1A1	HLA-A2[58]	
AFP	HLA-A2[59]	HLA-DR13[60]
ART-4	HLA-A24[61]	
BCLX (L)	HLA-A2[62]	
BING-4	HLA-A2[63]	
CALCA	HLA-A2[64]	
CD45	HLA-A24[65]	
CD274	HLA-A2[66]	
CLCA2	HLA-A2[67]	
CPSF	HLA-A2[68]	
Cyclin D1	HLA-A2[69]	HLA-DR4[70]
Cyp-B	HLA-A24[71]	
DKK1	HLA-A2[72]	
ENAH (hMena)	HLA-A2[73]	
EpCAM	HLA-A24[74]	
EphA3		HLA-DR11[75]
EZH2	HLA-A2[76]	
	HLA-A24[77]	
FGF-5	HLA-A3[78]	
HST-2(FGF-6)	HLA-A31[79]	
G250	HLA-A2[80]	
Glypican-3	HLA-A2[81]	
	HLA-A24[81]	
HEPACAM	HLA-A2[82]	

Antigen	MHC-I	MHC-II
Hepsin	HLA-A2[83]	
Her-2/neu	HLA-A2[84]	HLA-DR11[86]
	HLA-A3[4]	
	HLA-A24[85]	
HLA-DOB	HLA-A2[87]	
HLA-G		HLA-DR15[88]
		HLA-DR53[88]
HSPH1	HLA-A2[89]	
	HLA-A24[89]	
iCE	HLA-B7[90]	
IDO1	HLA-A2[91]	
IGF2B3	HLA-A2[92]	
	HLA-A3[92]	
IL13Ralpha2	HLA-A2[93]	
IMP-3		HLA-DR9[94]
		HLA-DR53[94]
Kallikrein 4	HLA-A2[95]	HLA-DP4[96]
		HLA-DR4[96]
		HLA-DR7[96]
KIF20A	HLA-A2[97]	
Lengsin	HLA-A2[98]	
Livin(ML-IAP)	HLA-A2[99]	
M-CSF	HLA-B35[100]	
MCSP		HLA-DR11[101]
Mdm-2	HLA-A2[102]	
Meloe	HLA-A2[103]	HLA-DQ2[104]
		HLA-DQ6[105]

Antigen	MHC-I	MHC-II
		HLA-DR1[104]
		HLA-DR11[105]
Midkine	HLA-A2[106]	HLA-DR4[107]
Mmp-2	HLA-A2[108]	
Mmp-7	HLA-A3[109]	
MUC-1	HLA-A2[110]	HLA-DR3[111]
MUC5AC	HLA-A24[112]	
NA17	HLA-A2[113]	
Nectin-4	HLA-A2[114]	
p15	HLA-A24[115]	
p53	HLA-A2[116]	HLA-DP5[119]
	HLA-A24[117]	HLA-DR4[120]
	HLA-B46[118]	HLA-DR14[119]
PAX5	HLA-A2[121]	
PBF	HLA-B55[122]	
PLAC1	HLA-A2[123]	
PRAME	HLA-A2[124]	
	HLA-A24[125]	
PSMA	HLA-A2[126]	HLA-DR3[128]
	HLA-A24[127]	HLA-DR4[128]
		HLA-DR7[128]
		HLA-DR11[128]
RAGE-1	HLA-B7[129]	
RGS5	HLA-A2[130]	
	HLA-A3[130]	
RHOC	HLA-A3[131]	
RNF43	HLA-A2[132]	

Antigen	MHC-I	MHC-II
	HLA-A24[132]	
RU1	HLA-B51[133]	
RU2AS	HLA-B7[134]	
SART-1	HLA-A24[135]	
	HLA-A26[136]	
SART-2	HLA-A24[137]	
SART-3	HLA-A10[138]	
	HLA-A24[139]	
Secrenin 1	HLA-A2[140]	
SOX-10	HLA-A2[40]	
STEAP1	HLA-A2[141]	
survivin	HLA-A2[142]	
Survivin-2B	HLA-A24[143]	
Telomerase	HLA-A2[144]	HLA-DR7[145]
		HLA-DR11[146]
TPBG	HLA-A2[147]	
TRG	HLA-B52[148]	
	HLA-B62[148]	
VEGF	HLA-B27[149]	
WT1	HLA-A2[150]	HLA-DP5[152]
	HLA-A24[151]	HLA-DR4[153]

Table S3. Tumor-specific antigens.

Antigen	MHC-I	MHC-II
BAGE-1	HLA-Cw16[154]	
CT37/FMR1NB	HLA-A2[155]	
Cyclin-A1	HLA-A2[156]	
DAM-6	HLA-A2[157]	
D393-CD20n		HLA-DR4[158]
GAGE-1/2/8	HLA-Cw6[159, 160]	
GAGE-3/4/5/6/7	HLA-A29[160]	
GnT-V(intron)	HLA-A2[161]	
HERV-E	HLA-A11[162]	
HERV-K-MEL	HLA-A2[163]	
KK-LC-1	HLA-B15[164]	
KM-HN-1	HLA-A24[165]	
LAGE-1	HLA-A2[166]	HLA-DP4[170]
	HLA-A31[167]	HLA-DR3[171]
	HLA-A68[168]	HLA-DR4[172]
	HLA-B7[169]	HLA-DR11[173]
		HLA-DR12[173]
		HLA-DR15[174]
LRPAP1	HLA-A2[175]	
LY6K	HLA-A24[176]	HLA-DP5[177]
		HLA-DR15[177]
MAGE-A1	HLA-A1[178]	HLA-DP4[189]
	HLA-A2[179]	HLA-DR13[190]
	HLA-A3[180]	HLA-DR15[191]
	HLA-A24[181]	

Antigen	MHC-I	MHC-II
	HLA-A28[182]	
	HLA-A68[182]	
	HLA-B7[183]	
	HLA-B35[18, 182]	
	HLA-B37[184]	
	HLA-B44[185]	
	HLA-B53[182]	
	HLA-B57[186]	
	HLA-Cw2[182]	
	HLA-Cw3[182]	
	HLA-Cw7[187]	
	HLA-Cw16[188]	
MAGE-A2	HLA-A2[192]	HLA-DR13[190]
	HLA-A3[180]	
	HLA-A24[193]	
	HLA-B37[184]	
	HLA-Cw7[194]	
MAGE-A3	HLA-A1[195]	HLA-DP4[203]
	HLA-A2[196]	HLA-DQ6[204]
	HLA-A3[180]	HLA-DR1[205]
	HLA-A24[197]	HLA-DR4[206]
	HLA-B18[198]	HLA-DR7[206]
	HLA-B35[199]	HLA-DR11[207]
	HLA-B37[184]	HLA-DR13[190]
	HLA-B40[200]	
	HLA-B44[201]	
	HLA-B52[202]	

Antigen	MHC-I	MHC-II
	HLA-Cw7[194]	
MAGE-A4	HLA-A1[208]	
	HLA-A2[209]	
	HLA-A24[210]	
	HLA-B37[211]	
MAGE-A6	HLA-A34[212]	HLA-DR4[215]
	HLA-B53[182]	HLA-DR13[182]
	HLA-B37[213]	
	HLA-Cw7[194]	
	HLA-Cw16[214]	
MAGE-A9	HLA-A2[216]	
MAGE-A10	HLA-A2[217]	
	HLA-B53[182]	
MAGE-A12	HLA-A2[196]	HLA-DP4[189]
	HLA-Cw7[218]	
MAGE-C1	HLA-A2[219]	HLA-DQ6[220]
		HLA-DR15[220]
MAGE-C2	HLA-A2[221]	HLA-DR15[224]
	HLA-B44[222]	
	HLA-B57[223]	
NA88-A	HLA-B13[225]	
NY-ES0-1	HLA-A2[226]	HLA-DP4[170]
	HLA-A24[227]	HLA-DR1[233]
	HLA-A31[167]	HLA-DR3[233]
	HLA-A68[228]	HLA-DR4[233]
	HLA-B7[229]	HLA-DR7[233]

Antigen	MHC-I	MHC-II
	HLA-B35[227]	HLA-DR8[234]
	HLA-B49[230]	HLA-DR9[234]
	HLA-B51[231]	HLA-DR15[174]
	HLA-B52[227]	HLA-DR52b[235]
	HLA-C12[227]	
	HLA-Cw3[232]	
	HLA-Cw6[232]	
SAGE	HLA-A24[210]	
Sp17	HLA-A1[236]	
SSX-2	HLA-A2[237]	HLA-DP1[238]
		HLA-DR1[239]
		HLA-DR3[240]
		HLA-DR4[241]
		HLA-DR11[242]
SSX-4		HLA-DP10[243]
		HLA-DR3[243]
		HLA-DR8[244]
		HLA-DR11[243]
		HLA-DR15[243]
		HLA-DR52[244]
TAG-1	HLA-A2[245]	
	HLA-B8[245]	
TAG-2	HLA-B8[245]	
TRAG-3	HLA-A2[246]	
TRP-2 (intron)	HLA-A68[247]	
XAGE-1b/GAGED2a	HLA-A2[248]	

Antigen	MHC-I	MHC-II
	HLA-DR4[248]	
	HLA-DR9[249]	

References

1. Harada M, Li YF, El-Gamil M, Rosenberg SA, Robbins PF. Use of an in Vitro Immunoselected Tumor Line to Identify Shared Melanoma Antigens Recognized by HLA-A*0201-restricted T Cells. *Cancer Research* 61(3), 1089 (2001).
2. Tsang KY, Zaremba S, Nieroda CA, Zhu MZ, Hamilton JM, Schlom J. Generation of Human Cytotoxic T Cells Specific for Human Carcinoembryonic Antigen Epitopes From Patients Immunized With Recombinant Vaccinia-CEA Vaccine. *JNCI: Journal of the National Cancer Institute* 87(13), 982-990 (1995).
3. Ras E, Van Der Burg SH, Zegveld ST *et al*. Identification of Potential HLA-A *0201 Restricted CTL Epitopes Derived from the Epithelial Cell Adhesion Molecule (Ep-CAM) and the Carcinoembryonic Antigen (CEA). *Human Immunology* 53(1), 81-89 (1997).
4. Kawashima I, Tsai V, Southwood S, Takesako K, Sette A, Celis E. Identification of HLA-A3-restricted Cytotoxic T Lymphocyte Epitopes from Carcinoembryonic Antigen and HER-2/neu by Primary in VitroImmunization with Peptide-pulsed Dendritic Cells. *Cancer Research* 59(2), 431-435 (1999).
5. Bremers AJA, Van Der Burg SH, Kuppen PJK, Kast WM, Van De Velde CJH, Melief CJM. The Use of Epstein-Barr Virus-Transformed B Lymphocyte Cell Lines in a Peptide-Reconstitution Assay: Identification of CEA-Related HLA-A*0301-Restricted Potential Cytotoxic T-Lymphocyte Epitopes. *Journal of Immunotherapy* 18(2), 77-85 (1995).
6. Nukaya I, Yasumoto M, Iwasaki T *et al*. Identification of HLA-A24 epitope peptides of carcinoembryonic antigen which induce tumor-reactive cytotoxic T lymphocyte. *International Journal of Cancer* 80(1), 92-97 (1999).
7. Huarte E, Sarobe P, Lasarte JJ *et al*. Identification of HLA-B27-restricted cytotoxic T lymphocyte epitope from carcinoembryonic antigen. *International Journal of Cancer* 97(1), 58-63 (2002).
8. Crosti M, Longhi R, Consogno G, Melloni G, Zannini P, Protti MP. Identification of Novel Subdominant Epitopes on the Carcinoembryonic Antigen Recognized by CD4+ T Cells of Lung Cancer Patients. *The Journal of Immunology* 176(8), 5093 (2006).
9. Ruiz M, Kobayashi H, Lasarte JJ *et al*. Identification and Characterization of a T-Helper Peptide from Carcinoembryonic Antigen. *Clinical Cancer Research* 10(8), 2860 (2004).
10. Kobayashi H, Omiya R, Ruiz M *et al*. Identification of an Antigenic Epitope for Helper T Lymphocytes from Carcinoembryonic Antigen. *Clinical Cancer Research* 8(10), 3219 (2002).
11. Campi G, Crosti M, Consogno G *et al*. CD4+ T Cells from Healthy Subjects and Colon Cancer Patients Recognize a Carcinoembryonic Antigen-specific Immunodominant Epitope. *Cancer Research* 63(23), 8481-8486 (2003).
12. Kawakami Y, Eliyahu S, Delgado CH *et al*. Identification of a human melanoma antigen recognized by tumor-infiltrating lymphocytes associated with in vivo tumor rejection. *Proceedings of the National Academy of Sciences* 91(14), 6458-6462 (1994).

13. Bakker AB, Schreurs MW, Tafazzul G *et al*. Identification of a novel peptide derived from the melanocyte-specific gp100 antigen as the dominant epitope recognized by an HLA-A2.1-restricted anti-melanoma CTL line. *Int J Cancer* 62(1), 97-102 (1995).
14. Kawashima I, Tsai V, Southwood S, Takesako K, Celis E, Sette A. Identification of GP100-derived, melanoma-specific cytotoxic T-lymphocyte epitopes restricted by HLA-A3 supertype molecules by primary in vitro immunization with peptide-pulsed dendritic cells. *International Journal of Cancer* 78(4), 518-524 (1998).
15. Robbins PF, El-Gamil M, Li YF, Fitzgerald EB, Kawakami Y, Rosenberg SA. The intronic region of an incompletely spliced gp100 gene transcript encodes an epitope recognized by melanoma-reactive tumor-infiltrating lymphocytes. *The Journal of Immunology* 159(1), 303 (1997).
16. Sensi M, Pellegatta S, Vegetti C, Nicolini G, Parmiani G, Anichini A. Identification of a novel gp100/pMel17 peptide presented by HLA-A*6801 and recognized on human melanoma by cytolytic T cell clones. *Tissue Antigens* 59(4), 273-279 (2002).
17. Lennerz V, Fatho M, Gentilini C *et al*. The response of autologous T cells to a human melanoma is dominated by mutated neoantigens. *Proc Natl Acad Sci U S A* 102(44), 16013-16018 (2005).
18. Benlalam H, Linard B, Guilloux Y *et al*. Identification of Five New HLA-B*3501-Restricted Epitopes Derived from Common Melanoma-Associated Antigens, Spontaneously Recognized by Tumor-Infiltrating Lymphocytes. *The Journal of Immunology* 171(11), 6283 (2003).
19. Castelli C, Tarsini P, Mazzocchi A *et al*. Novel HLA-Cw8-Restricted T Cell Epitopes Derived from Tyrosinase-Related Protein-2 and gp100 Melanoma Antigens. *The Journal of Immunology* 162(3), 1739-1748 (1999).
20. Kobayashi H, Lu J, Celis E. Identification of Helper T-Cell Epitopes That Encompass or Lie Proximal to Cytotoxic T-Cell Epitopes in the gp100 Melanoma Tumor Antigen. *Cancer Research* 61(20), 7577-7584 (2001).
21. Li K, Adibzadeh M, Halder T *et al*. Tumour-specific MHC-class-II-restricted responses after in vitro sensitization to synthetic peptides corresponding to gp100 and Annexin II eluted from melanoma cells. *Cancer Immunology, Immunotherapy* 47(1), 32-38 (1998).
22. Touloukian CE, Leitner WW, Topalian SL *et al*. Identification of a MHC class II-restricted human gp100 epitope using DR4-IE transgenic mice. *Journal of immunology (Baltimore, Md. : 1950)* 164(7), 3535-3542 (2000).
23. Lapointe R, Royal RE, Reeves ME, Altomare I, Robbins PF, Hwu P. Retrovirally Transduced Human Dendritic Cells Can Generate T Cells Recognizing Multiple MHC Class I and Class II Epitopes from the Melanoma Antigen Glycoprotein 100. *The Journal of Immunology* 167(8), 4758 (2001).
24. Jaramillo A, Majumder K, Manna PP *et al*. Identification of HLA-A3-restricted CD8+ T cell epitopes derived from mammaglobin-A, a tumor-associated antigen of human breast cancer. *International Journal of Cancer* 102(5), 499-506 (2002).
25. Kawakami Y, Eliyahu S, Sakaguchi K *et al*. Identification of the immunodominant peptides of the MART-1 human melanoma antigen recognized by the majority of HLA-A2-restricted tumor infiltrating lymphocytes. *The Journal of experimental medicine* 180(1), 347-352 (1994).
26. Kawakami Y, Eliyahu S, Delgado CH *et al*. Cloning of the gene coding for a shared human melanoma antigen recognized by autologous T cells infiltrating into tumor. *Proc Natl Acad Sci U S A* 91(9), 3515-3519 (1994).
27. Schneider J, Brichard V, Boon T, Meyer Zum Büschenfelde K-H, Wölfel T. Overlapping peptides of melanocyte differentiation antigen melan-A/MART-1 recognized by autologous cytolytic T lymphocytes in association with HLA-B45.1 and HLA-A2.1. *International Journal of Cancer* 75(3), 451-458 (1998).

28. Larrieu P, Renaud V, Godet Y, Jotereau F, Fonteneau J-F. A HLA-Cw*0701 restricted Melan-A/MART1 epitope presented by melanoma tumor cells to CD8+ tumor infiltrating lymphocytes. *Cancer Immunology, Immunotherapy* 57(5), 745-752 (2008).
29. Bioley G, Jandus C, Tuyaerts S *et al*. Melan-A/MART-1-Specific CD4 T Cells in Melanoma Patients: Identification of New Epitopes and Ex Vivo Visualization of Specific T Cells by MHC Class II Tetramers. *The Journal of Immunology* 177(10), 6769 (2006).
30. Zarour HM, Kirkwood JM, Kierstead LS *et al*. Melan-A/MART-1(51-73) represents an immunogenic HLA-DR4-restricted epitope recognized by melanoma-reactive CD4(+) T cells. *Proc Natl Acad Sci U S A* 97(1), 400-405 (2000).
31. Godefroy E, Scotto L, Souleimanian NE *et al*. Identification of two Melan-A CD4+ T cell epitopes presented by frequently expressed MHC class II alleles. *Clinical Immunology* 121(1), 54-62 (2006).
32. Salazar-Onfray F, Nakazawa T, Chhajlani V *et al*. Synthetic Peptides Derived from the Melanocyte-stimulating Hormone Receptor MC1R Can Stimulate HLA-A2-restricted Cytotoxic T Lymphocytes That Recognize Naturally Processed Peptides on Human Melanoma Cells. *Cancer Research* 57(19), 4348 (1997).
33. Wang W, Epler J, Salazar LG, Riddell SR. Recognition of Breast Cancer Cells by CD8+ Cytotoxic T-Cell Clones Specific for NY-BR-1. *Cancer Research* 66(13), 6826 (2006).
34. Touloukian CE, Leitner WW, Schnur RE *et al*. Normal tissue depresses while tumor tissue enhances human T cell responses in vivo to a novel self/tumor melanoma antigen, OA1. *Journal of immunology (Baltimore, Md. : 1950)* 170(3), 1579-1585 (2003).
35. Olson BM, Frye TP, Johnson LE *et al*. HLA-A2-restricted T-cell epitopes specific for prostatic acid phosphatase. *Cancer immunology, immunotherapy : CII* 59(6), 943-953 (2010).
36. Correale P, Nieroda C, Zaremba S *et al*. In Vitro Generation of Human Cytotoxic T Lymphocytes Specific for Peptides Derived From Prostate-Specific Antigen. *JNCI: Journal of the National Cancer Institute* 89(4), 293-300 (1997).
37. Corman JM, Sercarz EE, Nanda NK. Recognition of prostate-specific antigenic peptide determinants by human CD4 and CD8 T cells. *Clinical and experimental immunology* 114(2), 166-172 (1998).
38. Touloukian CE, Leitner WW, Robbins PF, Rosenberg SA, Restifo NP. Mining the Melanosome for Tumor Vaccine Targets. *Cancer Research* 61(22), 8100 (2001).
39. Walton SM, Gerlinger M, De La Rosa O *et al*. Spontaneous CD8 T Cell Responses against the Melanocyte Differentiation Antigen RAB38/NY-MEL-1 in Melanoma Patients. *The Journal of Immunology* 177(11), 8212 (2006).
40. Khong HT, Rosenberg SA. The Waardenburg syndrome type 4 gene, SOX10, is a novel tumor-associated antigen identified in a patient with a dramatic response to immunotherapy. *Cancer research* 62(11), 3020-3023 (2002).
41. Wang RF, Parkhurst MR, Kawakami Y, Robbins PF, Rosenberg SA. Utilization of an alternative open reading frame of a normal gene in generating a novel human cancer antigen. *The Journal of experimental medicine* 183(3), 1131-1140 (1996).
42. Touloukian CE, Leitner WW, Robbins PF *et al*. Expression of a "self-"antigen by human tumor cells enhances tumor antigen-specific CD4(+) T-cell function. *Cancer research* 62(18), 5144-5147 (2002).
43. Robbins PF, El-Gamil M, Li YF, Zeng G, Dudley M, Rosenberg SA. Multiple HLA class II-restricted melanocyte differentiation antigens are recognized by tumor-infiltrating lymphocytes from a patient with melanoma. *Journal of immunology (Baltimore, Md. : 1950)* 169(10), 6036-6047 (2002).

44. Osen W, Soltek S, Song M *et al*. Screening of human tumor antigens for CD4 T cell epitopes by combination of HLA-transgenic mice, recombinant adenovirus and antigen peptide libraries. *PloS one* 5(11), e14137-e14137 (2010).
45. Parkhurst MR, Fitzgerald EB, Southwood S, Sette A, Rosenberg SA, Kawakami Y. Identification of a Shared HLA-A*0201-restricted T-Cell Epitope from the Melanoma Antigen Tyrosinase-related Protein 2 (TRP2). *Cancer Research* 58(21), 4895 (1998).
46. Wang R-F, Johnston SL, Southwood S, Sette A, Rosenberg SA. Recognition of an Antigenic Peptide Derived from Tyrosinase-Related Protein-2 by CTL in the Context of HLA-A31 and -A33. *The Journal of Immunology* 160(2), 890 (1998).
47. Paschen A, Song M, Osen W *et al*. Detection of Spontaneous CD4+ T-Cell Responses in Melanoma Patients against a Tyrosinase-Related Protein-2–Derived Epitope Identified in HLA-DRB1*0301 Transgenic Mice. *Clinical Cancer Research* 11(14), 5241 (2005).
48. Kawakami Y, Robbins PF, Wang X *et al*. Identification of New Melanoma Epitopes on Melanosomal Proteins Recognized by Tumor Infiltrating T Lymphocytes Restricted by HLA-A1, -A2, and -A3 Alleles. *The Journal of Immunology* 161(12), 6985 (1998).
49. Wölfel T, Van Pel A, Brichard V *et al*. Two tyrosinase nonapeptides recognized on HLA-A2 melanomas by autologous cytolytic T lymphocytes. *European Journal of Immunology* 24(3), 759-764 (1994).
50. Brichard V, Van Pel A, Wölfel T *et al*. The tyrosinase gene codes for an antigen recognized by autologous cytolytic T lymphocytes on HLA-A2 melanomas. *The Journal of experimental medicine* 178(2), 489-495 (1993).
51. Kang X, Kawakami Y, El-Gamil M *et al*. Identification of a tyrosinase epitope recognized by HLA-A24-restricted, tumor-infiltrating lymphocytes. *The Journal of Immunology* 155(3), 1343 (1995).
52. Morel S, Ooms A, Van Pel A *et al*. A tyrosinase peptide presented by HLA-B35 is recognized on a human melanoma by autologous cytotoxic T lymphocytes. *International Journal of Cancer* 83(6), 755-759 (1999).
53. Brichard VG, Herman J, Van Pel A *et al*. A tyrosinase nonapeptide presented by HLA-B44 is recognized on a human melanoma by autologous cytolytic T lymphocytes. *European Journal of Immunology* 26(1), 224-230 (1996).
54. Topalian SL, Gonzales MI, Parkhurst M *et al*. Melanoma-specific CD4+ T cells recognize nonmutated HLA-DR-restricted tyrosinase epitopes. *The Journal of experimental medicine* 183(5), 1965-1971 (1996).
55. Kobayashi H, Kokubo T, Sato K *et al*. CD4+ T Cells from Peripheral Blood of a Melanoma Patient Recognize Peptides Derived from Nonmutated Tyrosinase. *Cancer Research* 58(2), 296 (1998).
56. Schmidt SM, Schag K, Müller MR *et al*. Induction of Adipophilin-Specific Cytotoxic T Lymphocytes Using a Novel HLA-A2-Binding Peptide That Mediates Tumor Cell Lysis. *Cancer Research* 64(3), 1164 (2004).
57. Harada M, Li YF, El-Gamil M, Ohnmacht GA, Rosenberg SA, Robbins PF. Melanoma-Reactive CD8 + T Cells Recognize a Novel Tumor Antigen Expressed in a Wide Variety of Tumor Types. *Journal of Immunotherapy* 24(4), (2001).
58. Visus C, Ito D, Amoscato A *et al*. Identification of Human Aldehyde Dehydrogenase 1 Family Member A1 as a Novel CD8+ T-Cell–Defined Tumor Antigen in Squamous Cell Carcinoma of the Head and Neck. *Cancer Research* 67(21), 10538 (2007).
59. Butterfield LH, Koh A, Meng W *et al*. Generation of Human T-cell Responses to an HLA-A2.1-restricted Peptide Epitope Derived from α-Fetoprotein. *Cancer Research* 59(13), 3134-3142 (1999).

60. Alisa A, Ives A, Pathan AA *et al*. Analysis of CD4+ T-Cell Responses to a Novel α-Fetoprotein-Derived Epitope in Hepatocellular Carcinoma Patients. *Clinical Cancer Research* 11(18), 6686-6694 (2005).
61. Kawano K, Gomi S, Tanaka K *et al*. Identification of a New Endoplasmic Reticulum-resident Protein Recognized by HLA-A24-restricted Tumor-infiltrating Lymphocytes of Lung Cancer. *Cancer Research* 60(13), 3550 (2000).
62. Sørensen RB, Hadrup SR, Køllgaard T, Svane IM, Thor Straten P, Andersen MH. Efficient tumor cell lysis mediated by a Bcl-X(L) specific T cell clone isolated from a breast cancer patient. *Cancer Immunology, Immunotherapy* 56(4), 527-533 (2007).
63. Rosenberg SA, Tong-On P, Li Y *et al*. Identification of BING-4 cancer antigen translated from an alternative open reading frame of a gene in the extended MHC class II region using lymphocytes from a patient with a durable complete regression following immunotherapy. *Journal of immunology (Baltimore, Md. : 1950)* 168(5), 2402-2407 (2002).
64. Durgeau A, Virk Y, Gros G *et al*. Human preprocalcitonin self-antigen generates TAP-dependent and -independent epitopes triggering optimised T-cell responses toward immune-escaped tumours. *Nature communications* 9(1), 5097-5097 (2018).
65. Tomita Y, Imai K, Senju S *et al*. A novel tumor-associated antigen, cell division cycle 45-like can induce cytotoxic T-lymphocytes reactive to tumor cells. *Cancer Science* 102(4), 697-705 (2011).
66. Munir S, Andersen GH, Met Ö *et al*. HLA-Restricted CTL That Are Specific for the Immune Checkpoint Ligand PD-L1 Occur with High Frequency in Cancer Patients. *Cancer Research* 73(6), 1764 (2013).
67. Konopitzky R, König U, Meyer RG, Sommergruber W, Wölfel T, Schweighoffer T. Identification of HLA-A*0201-Restricted T Cell Epitopes Derived from the Novel Overexpressed Tumor Antigen Calcium-Activated Chloride Channel 2. *The Journal of Immunology* 169(1), 540 (2002).
68. Maeda Y, Ito M, Harashima N *et al*. Cleavage and polyadenylation specificity factor (CPSF)-derived peptides can induce HLA-A2-restricted and tumor-specific CTLs in the majority of gastrointestinal cancer patients. *International Journal of Cancer* 99(3), 409-417 (2002).
69. Kondo E, Maecker B, Weihrauch MR *et al*. Cyclin D1–Specific Cytotoxic T Lymphocytes Are Present in the Repertoire of Cancer Patients: Implications for Cancer Immunotherapy. *Clinical Cancer Research* 14(20), 6574 (2008).
70. Dengjel J, Decker P, Schoor O *et al*. Identification of a naturally processed cyclin D1 T-helper epitope by a novel combination of HLA class II targeting and differential mass spectrometry. *European Journal of Immunology* 34(12), 3644-3651 (2004).
71. Gomi S, Nakao M, Niiya F *et al*. A Cyclophilin B Gene Encodes Antigenic Epitopes Recognized by HLA-A24-Restricted and Tumor-Specific CTLs. *The Journal of Immunology* 163(9), 4994 (1999).
72. Qian J, Xie J, Hong S *et al*. Dickkopf-1 (DKK1) is a widely expressed and potent tumor-associated antigen in multiple myeloma. *Blood* 110(5), 1587-1594 (2007).
73. Di Modugno F, Bronzi G, Scanlan MJ *et al*. Human mena protein, a serex-defined antigen overexpressed in breast cancer eliciting both humoral and CD8+ T-cell immune response. *International Journal of Cancer* 109(6), 909-918 (2004).
74. Tajima K, Demachi A, Ito Y *et al*. Identification of an epitope from the epithelial cell adhesion molecule eliciting HLA-A*2402-restricted cytotoxic T-lymphocyte responses. *Tissue Antigens* 64(6), 650-659 (2004).
75. Chiari R, Hames G, Stroobant V *et al*. Identification of a Tumor-specific Shared Antigen Derived From an Eph Receptor and Presented to CD4 T Cells on HLA Class II Molecules. *Cancer Research* 60(17), 4855 (2000).

76. Itoh Y, Komohara Y, Komatsu N *et al*. New peptides of the polycomb group protein enhancer of zeste homolog 2 with the potential to induce cancer-reactive cytotoxic T lymphocytes in human leukocyte antigen-A2+ prostate cancer patients. *Oncol Rep* 18(5), 1231-1237 (2007).
77. Ogata R, Matsueda S, Yao A, Noguchi M, Itoh K, Harada M. Identification of polycomb group protein enhancer of zeste homolog 2 (EZH2)-derived peptides immunogenic in HLA-A24+ prostate cancer patients. *The Prostate* 60(4), 273-281 (2004).
78. Hanada K-I, Yewdell JW, Yang JC. Immune recognition of a human renal cancer antigen through post-translational protein splicing. *Nature* 427(6971), 252-256 (2004).
79. Suzuki K, Sahara H, Okada Y *et al*. Identification of Natural Antigenic Peptides of a Human Gastric Signet Ring Cell Carcinoma Recognized by HLA-A31-Restricted Cytotoxic T Lymphocytes. *The Journal of Immunology* 163(5), 2783 (1999).
80. Vissers JLM, De Vries IJM, Schreurs MWJ *et al*. The Renal Cell Carcinoma-associated Antigen G250 Encodes a Human Leukocyte Antigen (HLA)-A2.1-restricted Epitope Recognized by Cytotoxic T Lymphocytes. *Cancer Research* 59(21), 5554 (1999).
81. Komori H, Nakatsura T, Senju S *et al*. Identification of HLA-A2- or HLA-A24-Restricted CTL Epitopes Possibly Useful for Glypican-3-Specific Immunotherapy of Hepatocellular Carcinoma. *Clinical Cancer Research* 12(9), 2689 (2006).
82. Neidert MC, Kowalewski DJ, Silginer M *et al*. The natural HLA ligandome of glioblastoma stem-like cells: antigen discovery for T cell-based immunotherapy. *Acta Neuropathologica* 135(6), 923-938 (2018).
83. Guo J, Li G, Tang J *et al*. HLA-A2-restricted Cytotoxic T Lymphocyte Epitopes from Human Hepsin as Novel Targets for Prostate Cancer Immunotherapy. *Scandinavian Journal of Immunology* 78(3), 248-257 (2013).
84. Rongcun Y, Salazar-Onfray F, Charo J *et al*. Identification of New HER2/neu Derived Peptide Epitopes That Can Elicit Specific CTL Against Autologous and Allogeneic Carcinomas and Melanomas. *The Journal of Immunology* 163(2), 1037 (1999).
85. Tanaka H, Tsunoda T, Nukaya I *et al*. Mapping the HLA-A24-restricted T-cell epitope peptide from a tumour-associated antigen HER2 / neu: possible immunotherapy for colorectal carcinomas. *British journal of cancer* 84(1), 94-99 (2001).
86. Anderson BW, Kudelka AP, Honda T *et al*. Induction of determinant spreading and of Th1 responses by in vitro stimulation with HER-2 peptides. *Cancer Immunology, Immunotherapy* 49(9), 459-468 (2000).
87. Kang YJ, Zeng W, Song W *et al*. Identification of human leucocyte antigen (HLA)-A*0201-restricted cytotoxic T lymphocyte epitopes derived from HLA-DOβ as a novel target for multiple myeloma. *British journal of haematology* 163(3), 343-351 (2013).
88. Ishibashi K, Kumai T, Ohkuri T *et al*. Epigenetic modification augments the immunogenicity of human leukocyte antigen G serving as a tumor antigen for T cell-based immunotherapy. *Oncoimmunology* 5(6), e1169356-e1169356 (2016).
89. Shimizu Y, Yoshikawa T, Kojima T *et al*. Heat shock protein 105 peptide vaccine could induce antitumor immune reactions in a phase I clinical trial. *Cancer science* 110(10), 3049-3060 (2019).
90. Ronsin C, Chung-Scott V, Poullion I, Aknouche N, Gaudin C, Triebel F. A Non-AUG-Defined Alternative Open Reading Frame of the Intestinal Carboxyl Esterase mRNA Generates an Epitope Recognized by Renal Cell Carcinoma-Reactive Tumor-Infiltrating Lymphocytes In Situ. *The Journal of Immunology* 163(1), 483 (1999).
91. Sørensen RB, Berge-Hansen L, Junker N *et al*. The immune system strikes back: cellular immune responses against indoleamine 2,3-dioxygenase. *PloS one* 4(9), e6910-e6910 (2009).

92. Tomita Y, Harao M, Senju S *et al*. Peptides derived from human insulin-like growth factor-II mRNA binding protein 3 can induce human leukocyte antigen-A2-restricted cytotoxic T lymphocytes reactive to cancer cells. *Cancer Science* 102(1), 71-78 (2011).
93. Okano F, Storkus WJ, Chambers WH, Pollack IF, Okada H. Identification of a Novel HLA-A*0201-restricted, Cytotoxic T Lymphocyte Epitope in a Human Glioma-associated Antigen, Interleukin 13 Receptor α2 Chain. *Clinical Cancer Research* 8(9), 2851-2855 (2002).
94. Hirayama M, Tomita Y, Yuno A *et al*. An oncofetal antigen, IMP-3-derived long peptides induce immune responses of both helper T cells and CTLs. *Oncoimmunology* 5(6), e1123368-e1123368 (2016).
95. Wilkinson R, Woods K, D'rozario R *et al*. Human kallikrein 4 signal peptide induces cytotoxic T cell responses in healthy donors and prostate cancer patients. *Cancer Immunology, Immunotherapy* 61(2), 169-179 (2012).
96. Hural JA, Friedman RS, Mcnabb A, Steen SS, Henderson RA, Kalos M. Identification of Naturally Processed CD4 T Cell Epitopes from the Prostate-Specific Antigen Kallikrein 4 Using Peptide-Based In Vitro Stimulation. *The Journal of Immunology* 169(1), 557-565 (2002).
97. Imai K, Hirata S, Irie A *et al*. Identification of HLA-A2-restricted CTL epitopes of a novel tumour-associated antigen, KIF20A, overexpressed in pancreatic cancer. *British journal of cancer* 104(2), 300-307 (2011).
98. Nakatsugawa M, Horie K, Yoshikawa T *et al*. Identification of an HLA-A*0201-restricted cytotoxic T lymphocyte epitope from the lung carcinoma antigen, Lengsin. *Int J Oncol* 39(4), 1041-1049 (2011).
99. Schmollinger JC, Vonderheide RH, Hoar KM *et al*. Melanoma inhibitor of apoptosis protein (ML-IAP) is a target for immune-mediated tumor destruction. *Proceedings of the National Academy of Sciences* 100(6), 3398-3403 (2003).
100. Probst-Kepper M, Stroobant V, Kridel R *et al*. An alternative open reading frame of the human macrophage colony-stimulating factor gene is independently translated and codes for an antigenic peptide of 14 amino acids recognized by tumor-infiltrating CD8 T lymphocytes. *The Journal of experimental medicine* 193(10), 1189-1198 (2001).
101. Erfurt C, Sun Z, Haendle I *et al*. Tumor-Reactive CD4+ T Cell Responses to the Melanoma-Associated Chondroitin Sulphate Proteoglycan in Melanoma Patients and Healthy Individuals in the Absence of Autoimmunity. *The Journal of Immunology* 178(12), 7703 (2007).
102. Asai T, Storkus WJ, Mueller-Berghaus J *et al*. In vitro generated cytolytic T lymphocytes reactive against head and neck cancer recognize multiple epitopes presented by HLA-A2, including peptides derived from the p53 and MDM-2 proteins. *Cancer Immunity Archive* 2(1), 3 (2002).
103. Godet Y, Moreau-Aubry A, Guilloux Y *et al*. MELOE-1 is a new antigen overexpressed in melanomas and involved in adoptive T cell transfer efficiency. *The Journal of experimental medicine* 205(11), 2673-2682 (2008).
104. Bobinet M, Vignard V, Rogel A *et al*. MELOE-1 antigen contains multiple HLA class II T cell epitopes recognized by Th1 CD4+ T cells from melanoma patients. *PloS one* 7(12), e51716-e51716 (2012).
105. Rogel A, Vignard V, Bobinet M, Labarriere N, Lang F. A long peptide from MELOE-1 contains multiple HLA class II T cell epitopes in addition to the HLA-A*0201 epitope: an attractive candidate for melanoma vaccination. *Cancer Immunology, Immunotherapy* 60(3), 327-337 (2011).
106. Kerzerho J, Adotevi O, Castelli FA *et al*. The Angiogenic Growth Factor and Biomarker Midkine Is a Tumor-Shared Antigen. *The Journal of Immunology* 185(1), 418 (2010).

107. Kerzerho J, Schneider A, Favry E, Castelli FA, Maillère B. The signal peptide of the tumor-shared antigen midkine hosts CD4+ T cell epitopes. *The Journal of biological chemistry* 288(19), 13370-13377 (2013).
108. Godefroy E, Moreau-Aubry A, Diez E, Dreno B, Jotereau F, Guilloux Y. alpha v beta3-dependent cross-presentation of matrix metalloproteinase-2 by melanoma cells gives rise to a new tumor antigen. *The Journal of experimental medicine* 202(1), 61-72 (2005).
109. Yokoyama Y, Grünebach F, Schmidt SM *et al*. Matrilysin (MMP-7) Is a Novel Broadly Expressed Tumor Antigen Recognized by Antigen-Specific T Cells. *Clinical Cancer Research* 14(17), 5503 (2008).
110. Brossart P, Heinrich KS, Stuhler G *et al*. Identification of HLA-A2–Restricted T-Cell Epitopes Derived From the MUC1 Tumor Antigen for Broadly Applicable Vaccine Therapies. *Blood* 93(12), 4309-4317 (1999).
111. Hiltbold EM, Ciborowski P, Finn OJ. Naturally Processed Class II Epitope from the Tumor Antigen MUC1 Primes Human CD4+ T Cells. *Cancer Research* 58(22), 5066 (1998).
112. Yamazoe S, Tanaka H, Iwauchi T *et al*. Identification of HLA-A*0201- and A*2402-Restricted Epitopes of Mucin 5AC Expressed in Advanced Pancreatic Cancer. *Pancreas* 40(6), 896-904 (2011).
113. Labarrière N, Bretaudeau L, Gervois N *et al*. Apoptotic body–loaded dendritic cells efficiently cross-prime cytotoxic T lymphocytes specific for NA17-A antigen but not for Melan-A/MART-1 antigen. *International Journal of Cancer* 101(3), 280-286 (2002).
114. Lopez M, Ghidouche A, Rochas C *et al*. Identification of a naturally processed HLA-A*02:01-restricted CTL epitope from the human tumor-associated antigen Nectin-4. *Cancer Immunology, Immunotherapy* 65(10), 1177-1188 (2016).
115. Robbins PF, El-Gamil M, Li YF *et al*. Cloning of a new gene encoding an antigen recognized by melanoma-specific HLA-A24-restricted tumor-infiltrating lymphocytes. *The Journal of Immunology* 154(11), 5944 (1995).
116. Röpke M, Hald J, Guldberg P *et al*. Spontaneous human squamous cell carcinomas are killed by a human cytotoxic T lymphocyte clone recognizing a wild-type p53-derived peptide. *Proc Natl Acad Sci U S A* 93(25), 14704-14707 (1996).
117. Eura M, Chikamatsu K, Katsura F *et al*. A Wild-type Sequence p53 Peptide Presented by HLA-A24 Induces Cytotoxic T Lymphocytes that Recognize Squamous Cell Carcinomas of the Head and Neck. *Clinical Cancer Research* 6(3), 979-986 (2000).
118. Azuma K, Shichijo S, Maeda Y *et al*. Mutated p53 Gene Encodes a Nonmutated Epitope Recognized by HLA-B*4601-restricted and Tumor Cell-reactive CTLs at Tumor Site. *Cancer Research* 63(4), 854 (2003).
119. Fujita H, Senju S, Yokomizo H *et al*. Evidence that HLA class II-restricted human CD4+ T cells specific to p53 self peptides respond to p53 proteins of both wild and mutant forms. *European Journal of Immunology* 28(1), 305-316 (1998).
120. Chikamatsu K, Albers A, Stanson J *et al*. p53 (110–124)-specific Human CD4+ T-helper Cells Enhance in Vitro Generation and Antitumor Function of Tumor-reactive CD8+ T Cells. *Cancer Research* 63(13), 3675 (2003).
121. Yan M, Himoudi N, Pule M *et al*. Development of Cellular Immune Responses against PAX5, a Novel Target for Cancer Immunotherapy. *Cancer Research* 68(19), 8058 (2008).
122. Tsukahara T, Nabeta Y, Kawaguchi S *et al*. Identification of Human Autologous Cytotoxic T-Lymphocyte-Defined Osteosarcoma Gene That Encodes a Transcriptional Regulator, Papillomavirus Binding Factor. *Cancer Research* 64(15), 5442 (2004).
123. Li Q, Liu M, Wu M *et al*. PLAC1-specific TCR-engineered T cells mediate antigen-specific antitumor effects in breast cancer. *Oncology letters* 15(4), 5924-5932 (2018).

124. Kessler JH, Beekman NJ, Bres-Vloemans SA *et al*. Efficient identification of novel HLA-A(*)0201-presented cytotoxic T lymphocyte epitopes in the widely expressed tumor antigen PRAME by proteasome-mediated digestion analysis. *The Journal of experimental medicine* 193(1), 73-88 (2001).
125. Ikeda H, Lethé B, Lehmann F *et al*. Characterization of an Antigen That Is Recognized on a Melanoma Showing Partial HLA Loss by CTL Expressing an NK Inhibitory Receptor. *Immunity* 6(2), 199-208 (1997).
126. Murphy G, Tjoa B, Ragde H, Kenny G, Boynton A. Phase I clinical trial: T-cell therapy for prostate cancer using autologous dendritic cells pulsed with HLA-A0201-specific peptides from prostate-specific membrane antigen. *The Prostate* 29(6), 371-380 (1996).
127. Horiguchi Y, Nukaya I, Okazawa K *et al*. Screening of HLA-A24-restricted Epitope Peptides from Prostate-specific Membrane Antigen That Induce Specific Antitumor Cytotoxic T Lymphocytes. *Clinical Cancer Research* 8(12), 3885 (2002).
128. Schroers R, Shen L, Rollins L *et al*. Identification of MHC Class II-restricted T-cell Epitopes in Prostate-specific Membrane Antigen. *Clinical Cancer Research* 9(9), 3260 (2003).
129. Gaugler B, Brouwenstijn N, Vantomme V *et al*. A new gene coding for an antigen recognized by autologous cytolytic T lymphocytes on a human renal carcinoma. *Immunogenetics* 44(5), 323-330 (1996).
130. Boß CN, Grünebach F, Brauer K *et al*. Identification and Characterization of T-Cell Epitopes Deduced from RGS5, a Novel Broadly Expressed Tumor Antigen. *Clinical Cancer Research* 13(11), 3347 (2007).
131. Wenandy L, Sørensen RB, Svane IM, Thor Straten P, Andersen MH. RhoC a new target for therapeutic vaccination against metastatic cancer. *Cancer Immunology, Immunotherapy* 57(12), 1871-1878 (2008).
132. Uchida N, Tsunoda T, Wada S, Furukawa Y, Nakamura Y, Tahara H. Ring Finger Protein 43 as a New Target for Cancer Immunotherapy. *Clinical Cancer Research* 10(24), 8577 (2004).
133. Morel S, Lévy F, Burlet-Schiltz O *et al*. Processing of Some Antigens by the Standard Proteasome but Not by the Immunoproteasome Results in Poor Presentation by Dendritic Cells. *Immunity* 12(1), 107-117 (2000).
134. Van Den Eynde BJ, Gaugler B, Probst-Kepper M *et al*. A new antigen recognized by cytolytic T lymphocytes on a human kidney tumor results from reverse strand transcription. *The Journal of experimental medicine* 190(12), 1793-1800 (1999).
135. Kikuchi M, Nakao M, Inoue Y *et al*. Identification of a SART-1-derived peptide capable of inducing HLA-A24-restricted and tumor-specific cytotoxic T lymphocytes. *International Journal of Cancer* 81(3), 459-466 (1999).
136. Shichijo S, Nakao M, Imai Y *et al*. A Gene Encoding Antigenic Peptides of Human Squamous Cell Carcinoma Recognized by Cytotoxic T Lymphocytes. *Journal of Experimental Medicine* 187(3), 277-288 (1998).
137. Nakao M, Shichijo S, Imaizumi T *et al*. Identification of a Gene Coding for a New Squamous Cell Carcinoma Antigen Recognized by the CTL. *The Journal of Immunology* 164(5), 2565 (2000).
138. Ito M, Shichijo S, Miyagi Y *et al*. Identification of SART3-derived peptides capable of inducing HLA-A2-restricted and tumor-specific CTLs in cancer patients with different HLA-A2 subtypes. *International Journal of Cancer* 88(4), 633-639 (2000).
139. Yang D, Nakao M, Shichijo S *et al*. Identification of a Gene Coding for a Protein Possessing Shared Tumor Epitopes Capable of Inducing HLA-A24-restricted Cytotoxic T Lymphocytes inCancer Patients. *Cancer Research* 59(16), 4056 (1999).

140. Suda T, Tsunoda T, Uchida N *et al*. Identification of secernin 1 as a novel immunotherapy target for gastric cancer using the expression profiles of cDNA microarray. *Cancer science* 97 411-419 (2006).
141. Rodeberg DA, Nuss RA, Elsawa SF, Celis E. Recognition of six-transmembrane epithelial antigen of the prostate-expressing tumor cells by peptide antigen-induced cytotoxic T lymphocytes. *Clinical cancer research : an official journal of the American Association for Cancer Research* 11(12), 4545-4552 (2005).
142. Schmitz M, Diestelkoetter P, Weigle B *et al*. Generation of Survivin-specific $CD8^+$ T Effector Cells by Dendritic Cells Pulsed with Protein or Selected Peptides. *Cancer Research* 60(17), 4845 (2000).
143. Hirohashi Y, Torigoe T, Maeda A *et al*. An HLA-A24-restricted Cytotoxic T Lymphocyte Epitope of a Tumor-associated Protein, Survivin. *Clinical Cancer Research* 8(6), 1731 (2002).
144. Vonderheide RH, Hahn WC, Schultze JL, Nadler LM. The Telomerase Catalytic Subunit Is a Widely Expressed Tumor-Associated Antigen Recognized by Cytotoxic T Lymphocytes. *Immunity* 10(6), 673-679 (1999).
145. Schroers R, Huang XF, Hammer J, Zhang J, Chen S-Y. Identification of HLA DR7-restricted Epitopes from Human Telomerase Reverse Transcriptase Recognized by CD4+ T-Helper Cells. *Cancer Research* 62(9), 2600 (2002).
146. Schroers R, Shen L, Rollins L *et al*. Human Telomerase Reverse Transcriptase-Specific T-Helper Responses Induced by Promiscuous Major Histocompatibility Complex Class II-Restricted Epitopes. *Clinical Cancer Research* 9(13), 4743 (2003).
147. Tykodi SS, Satoh S, Deming JD, Chou J, Harrop R, Warren EH. CD8+ T-cell clones specific for the 5T4 antigen target renal cell carcinoma tumor-initiating cells in a murine xenograft model. *Journal of immunotherapy (Hagerstown, Md. : 1997)* 35(7), 523-533 (2012).
148. Ohkouchi S, Kawamoto N, Koga M *et al*. Identification of a CTL-directed epitope encoded by an intron of the putative tumor suppressor gene Testin of the common fragile site 7G region: a peptide vaccine candidate for HLA-B52+ and HLA-62+ cancer patients. *European Journal of Immunology* 33(11), 2964-2973 (2003).
149. Weinzierl AO, Maurer D, Altenberend F *et al*. A Cryptic Vascular Endothelial Growth Factor T-Cell Epitope: Identification and Characterization by Mass Spectrometry and T-Cell Assays. *Cancer Research* 68(7), 2447 (2008).
150. Oka Y, Elisseeva OA, Tsuboi A *et al*. Human cytotoxic T-lymphocyte responses specific for peptides of the wild-type Wilms' tumor gene (WT1) product. *Immunogenetics* 51(2), 99-107 (2000).
151. Azuma T, Makita M, Ninomiya K, Fujita S, Harada M, Yasukawa M. Identification of a novel WT1-derived peptide which induces human leucocyte antigen-A24-restricted anti-leukaemia cytotoxic T lymphocytes. *British Journal of Haematology* 116(3), 601-603 (2002).
152. Guo Y, Niiya H, Azuma T *et al*. Direct recognition and lysis of leukemia cells by WT1-specific CD4+ T lymphocytes in an HLA class II-restricted manner. *Blood* 106(4), 1415-1418 (2005).
153. Knights A, Zaniou A, Rees RC, Pawelec G, Müller L. Prediction of an HLA-DR-binding peptide derived from Wilms' tumour 1 protein and demonstration of in vitro immunogenicity of WT1(124–138)-pulsed dendritic cells generated according to an optimised protocol. *Cancer Immunology, Immunotherapy* 51(5), 271-281 (2002).
154. Boël P, Wildmann C, Sensi ML *et al*. BAGE: a new gene encoding an antigen recognized on human melanomas by cytolytic T lymphocytes. *Immunity* 2(2), 167-175 (1995).
155. Flores-Villanueva PO, Ganachari M, Guio H, Mejia JA, Granados J. An Isolated TCR αβ Restricted by HLA-A*02:01/CT37 Peptide Redirecting CD8(+) T Cells To Kill and Secrete IFN-γ in Response to

Lung Adenocarcinoma Cell Lines. *Journal of immunology (Baltimore, Md. : 1950)* 200(8), 2965-2977 (2018).
156. Ochsenreither S, Majeti R, Schmitt T *et al*. Cyclin-A1 represents a new immunogenic targetable antigen expressed in acute myeloid leukemia stem cells with characteristics of a cancer-testis antigen. *Blood* 119(23), 5492-5501 (2012).
157. Fleischhauer K, Gattinoni L, Dalerba P *et al*. The DAM Gene Family Encodes a New Group of Tumor-specific Antigens Recognized by Human Leukocyte Antigen A2-restricted Cytotoxic T Lymphocytes. *Cancer Research* 58(14), 2969 (1998).
158. Vauchy C, Gamonet C, Ferrand C *et al*. CD20 alternative splicing isoform generates immunogenic CD4 helper T epitopes. *International Journal of Cancer* 137(1), 116-126 (2015).
159. Van Den Eynde B, Peeters O, De Backer O, Gaugler B, Lucas S, Boon T. A new family of genes coding for an antigen recognized by autologous cytolytic T lymphocytes on a human melanoma. *The Journal of experimental medicine* 182(3), 689-698 (1995).
160. De Backer O, Arden KC, Boretti M *et al*. Characterization of the GAGE Genes That Are Expressed in Various Human Cancers and in Normal Testis. *Cancer Research* 59(13), 3157 (1999).
161. Guilloux Y, Lucas S, Brichard VG *et al*. A peptide recognized by human cytolytic T lymphocytes on HLA-A2 melanomas is encoded by an intron sequence of the N-acetylglucosaminyltransferase V gene. *The Journal of experimental medicine* 183(3), 1173-1183 (1996).
162. Takahashi Y, Harashima N, Kajigaya S *et al*. Regression of human kidney cancer following allogeneic stem cell transplantation is associated with recognition of an HERV-E antigen by T cells. *The Journal of clinical investigation* 118(3), 1099-1109 (2008).
163. Schiavetti F, Thonnard J, Colau D, Boon T, Coulie PG. A Human Endogenous Retroviral Sequence Encoding an Antigen Recognized on Melanoma by Cytolytic T Lymphocytes. *Cancer Research* 62(19), 5510 (2002).
164. Fukuyama T, Hanagiri T, Takenoyama M *et al*. Identification of a New Cancer/Germline Gene, KK-LC-1, Encoding an Antigen Recognized by Autologous CTL Induced on Human Lung Adenocarcinoma. *Cancer Research* 66(9), 4922 (2006).
165. Monji M, Nakatsura T, Senju S *et al*. Identification of a Novel Human Cancer/Testis Antigen, KM-HN-1, Recognized by Cellular and Humoral Immune Responses. *Clinical Cancer Research* 10(18), 6047 (2004).
166. Aarnoudse CA, Van Den Doel PB, Heemskerk B, Schrier PI. Interleukin-2-induced, melanoma-specific T cells recognize camel, an unexpected translation product of LAGE-1. *International Journal of Cancer* 82(3), 442-448 (1999).
167. Wang R-F, Johnston SL, Zeng G, Topalian SL, Schwartzentruber DJ, Rosenberg SA. A Breast and Melanoma-Shared Tumor Antigen: T Cell Responses to Antigenic Peptides Translated from Different Open Reading Frames. *The Journal of Immunology* 161(7), 3596 (1998).
168. Sun Z, Lethé B, Zhang Y *et al*. A new LAGE-1 peptide recognized by cytolytic T lymphocytes on HLA-A68 tumors. *Cancer Immunology, Immunotherapy* 55(6), 644-652 (2006).
169. Slager EH, Van Der Minne CE, Goudsmit J *et al*. Induction of CAMEL/NY-ESO-ORF2-specific CD8+ T cells upon stimulation with dendritic cells infected with a modified Ad5 vector expressing a chimeric Ad5/35 fiber. *Cancer Gene Therapy* 11(3), 227-236 (2004).
170. Zeng G, Wang X, Robbins PF, Rosenberg SA, Wang RF. CD4(+) T cell recognition of MHC class II-restricted epitopes from NY-ESO-1 presented by a prevalent HLA DP4 allele: association with NY-ESO-1 antibody production. *Proc Natl Acad Sci U S A* 98(7), 3964-3969 (2001).
171. Slager EH, Van Der Minne CE, Krüse M, Krueger DD, Griffioen M, Osanto S. Identification of Multiple HLA-DR-Restricted Epitopes of the Tumor-Associated Antigen CAMEL by CD4+Th1/Th2 Lymphocytes. *The Journal of Immunology* 172(8), 5095 (2004).

172. Jäger E, Jäger D, Karbach J *et al*. Identification of NY-ESO-1 epitopes presented by human histocompatibility antigen (HLA)-DRB4*0101-0103 and recognized by CD4(+) T lymphocytes of patients with NY-ESO-1-expressing melanoma. *The Journal of experimental medicine* 191(4), 625-630 (2000).
173. Slager EH, Borghi M, Van Der Minne CE *et al*. CD4+ Th2 Cell Recognition of HLA-DR-Restricted Epitopes Derived from CAMEL: A Tumor Antigen Translated in an Alternative Open Reading Frame. *The Journal of Immunology* 170(3), 1490 (2003).
174. Hasegawa K, Noguchi Y, Koizumi F *et al*. In vitro Stimulation of CD8 and CD4 T Cells by Dendritic Cells Loaded with a Complex of Cholesterol-Bearing Hydrophobized Pullulan and NY-ESO-1 Protein: Identification of a New HLA-DR15–Binding CD4 T-Cell Epitope. *Clinical Cancer Research* 12(6), 1921 (2006).
175. Marijt KA, Blijleven L, Verdegaal EME *et al*. Identification of non-mutated neoantigens presented by TAP-deficient tumors. *The Journal of experimental medicine* 215(9), 2325-2337 (2018).
176. Suda T, Tsunoda T, Daigo Y, Nakamura Y, Tahara H. Identification of human leukocyte antigen-A24-restricted epitope peptides derived from gene products upregulated in lung and esophageal cancers as novel targets for immunotherapy. *Cancer Science* 98(11), 1803-1808 (2007).
177. Tomita Y, Yuno A, Tsukamoto H *et al*. Identification of immunogenic LY6K long peptide encompassing both CD4(+) and CD8(+) T-cell epitopes and eliciting CD4(+) T-cell immunity in patients with malignant disease. *Oncoimmunology* 3 e28100-e28100 (2014).
178. Van Der Bruggen P, Traversari C, Chomez P *et al*. A gene encoding an antigen recognized by cytolytic T lymphocytes on a human melanoma. *Science* 254(5038), 1643 (1991).
179. Pascolo S, Schirle M, Gückel B *et al*. A MAGE-A1 HLA-A*0201 Epitope Identified by Mass Spectrometry. *Cancer Research* 61(10), 4072 (2001).
180. Mcintyre CA, Rees RC, Platts KE *et al*. Identification of peptide epitopes of MAGE-1, -2, -3 that demonstrate HLA-A3-specific binding. *Cancer Immunology, Immunotherapy* 42(4), 246-250 (1996).
181. Fujie T, Tahara K, Tanaka F, Mori M, Takesako K, Akiyoshi T. A MAGE-1-encoded HLA-A24-binding synthetic peptide induces specific anti-tumor cytotoxic T lymphocytes. *International Journal of Cancer* 80(2), 169-172 (1999).
182. Chaux P, Luiten R, Demotte N *et al*. Identification of Five MAGE-A1 Epitopes Recognized by Cytolytic T Lymphocytes Obtained by In Vitro Stimulation with Dendritic Cells Transduced with MAGE-A1. *The Journal of Immunology* 163(5), 2928 (1999).
183. Luiten R, Van Der Bruggen P. A MAGE-A1 peptide is recognized on HLA-B7 human tumors by cytolytic T lymphocytes. *Tissue Antigens* 55(2), 149-152 (2000).
184. Tanzarella S, Russo V, Lionello I *et al*. Identification of a Promiscuous T-Cell Epitope Encoded by Multiple Members of the MAGE Family. *Cancer Research* 59(11), 2668-2674 (1999).
185. Stroobant V, Demotte N, Luiten RM *et al*. Inefficient exogenous loading of a tapasin-dependent peptide onto HLA-B*44:02 can be improved by acid treatment or fixation of target cells. *European journal of immunology* 42(6), 1417-1428 (2012).
186. Corbière V, Nicolay H, Russo V *et al*. Identification of a MAGE-1 peptide recognized by cytolytic T lymphocytes on HLA-B*5701 tumors. *Tissue Antigens* 63(5), 453-457 (2004).
187. Goodyear OC, Pearce H, Pratt G, Moss P. Dominant responses with conservation of T-cell receptor usage in the CD8+ T-cell recognition of a cancer testis antigen peptide presented through HLA-Cw7 in patients with multiple myeloma. *Cancer Immunology, Immunotherapy* 60(12), 1751-1761 (2011).
188. Bruggen PVD, Szikora J-P, Boël P *et al*. Autologous cytolytic T lymphocytes recognize a MAGE-1 nonapeptide on melanomas expressing HLA-Cw* 1601. *European Journal of Immunology* 24(9), 2134-2140 (1994).

189. Wang X-F, Cohen WM, Castelli FA *et al*. Selective identification of HLA-DP4 binding T cell epitopes encoded by the MAGE-A gene family. *Cancer Immunology, Immunotherapy* 56(6), 807-818 (2007).
190. Goodyear OC, Pratt G, Mclarnon A, Cook M, Piper K, Moss P. Differential pattern of CD4+ and CD8+ T-cell immunity to MAGE-A1/A2/A3 in patients with monoclonal gammopathy of undetermined significance (MGUS) and multiple myeloma. *Blood* 112(8), 3362-3372 (2008).
191. Chaux P, Lethé B, Van Snick J *et al*. A MAGE-1 peptide recognized on HLA-DR15 by CD4+ T cells. *European Journal of Immunology* 31(6), 1910-1916 (2001).
192. Visseren MJW, Van Der Burg SH, Van Der Voort EIH *et al*. Identification of HLA-A*0201-restricted CTL epitopes encoded by the tumor-specific MAGE-2 gene product. *International Journal of Cancer* 73(1), 125-130 (1997).
193. Tahara K, Takesako K, Sette A, Celis E, Kitano S, Akiyoshi T. Identification of a MAGE-2-encoded Human Leukocyte Antigen-A24-binding Synthetic Peptide That Induces Specific Antitumor Cytotoxic T Lymphocytes. *Clinical Cancer Research* 5(8), 2236 (1999).
194. Breckpot K, Heirman C, De Greef C, Van Der Bruggen P, Thielemans K. Identification of New Antigenic Peptide Presented by HLA-Cw7 and Encoded by Several MAGE Genes Using Dendritic Cells Transduced with Lentiviruses. *The Journal of Immunology* 172(4), 2232 (2004).
195. Gaugler B, Van Den Eynde B, Van Der Bruggen P *et al*. Human gene MAGE-3 codes for an antigen recognized on a melanoma by autologous cytolytic T lymphocytes. *The Journal of experimental medicine* 179(3), 921-930 (1994).
196. Van Der Bruggen P, Bastin J, Gajewski T *et al*. A peptide encoded by human gene MAGE-3 and presented by HLA-A2 induces cytolytic T lymphocytes that recognize tumor cells expressing MAGE-3. *European Journal of Immunology* 24(12), 3038-3043 (1994).
197. Tanaka F, Fujie T, Tahara K *et al*. Induction of Antitumor Cytotoxic T Lymphocytes with a MAGE-3-encoded Synthetic Peptide Presented by Human Leukocytes Antigen-A24. *Cancer Research* 57(20), 4465 (1997).
198. Bilsborough J, Panichelli C, Duffour MT *et al*. A MAGE-3 peptide presented by HLA-B44 is also recognized by cytolytic T lymphocytes on HLA-B18. *Tissue Antigens* 60(1), 16-24 (2002).
199. Schultz ES, Zhang Y, Knowles R *et al*. A MAGE-3 peptide recognized on HLA-B35 and HLA-A1 by cytolytic T lymphocytes. *Tissue Antigens* 57(2), 103-109 (2001).
200. Schultz ES, Chapiro J, Lurquin C *et al*. The production of a new MAGE-3 peptide presented to cytolytic T lymphocytes by HLA-B40 requires the immunoproteasome. *The Journal of experimental medicine* 195(4), 391-399 (2002).
201. Herman J, Van Der Bruggen P, Luescher IF *et al*. A peptide encoded by the human MAGE3 gene and presented by HLA-1344 induces cytolytic T lymphocytes that recognize tumor cells expressing MAGE3. *Immunogenetics* 43(6), 377-383 (1996).
202. Russo V, Tanzarella S, Dalerba P *et al*. Dendritic cells acquire the MAGE-3 human tumor antigen from apoptotic cells and induce a class I-restricted T cell response. *Proceedings of the National Academy of Sciences* 97(5), 2185 (2000).
203. Schultz ES, Lethé B, Cambiaso CL *et al*. A MAGE-A3 Peptide Presented by HLA-DP4 Is Recognized on Tumor Cells by CD4+ Cytolytic T Lymphocytes. *Cancer Research* 60(22), 6272 (2000).
204. Schultz ES, Schuler-Thurner B, Stroobant V *et al*. Functional Analysis of Tumor-Specific Th Cell Responses Detected in Melanoma Patients after Dendritic Cell-Based Immunotherapy. *The Journal of Immunology* 172(2), 1304 (2004).
205. Zhang Y, Chaux P, Stroobant V *et al*. A MAGE-3 Peptide Presented by HLA-DR1 to CD4+ T Cells That Were Isolated from a Melanoma Patient Vaccinated with a MAGE-3 Protein. *The Journal of Immunology* 171(1), 219 (2003).

206. Kobayashi H, Song Y, Hoon DSB, Appella E, Celis E. Tumor-reactive T Helper Lymphocytes Recognize a Promiscuous MAGE-A3 Epitope Presented by Various Major Histocompatibility Complex Class II Alleles. *Cancer Research* 61(12), 4773 (2001).
207. Manici S, Sturniolo T, Imro MA *et al*. Melanoma cells present a MAGE-3 epitope to CD4(+) cytotoxic T cells in association with histocompatibility leukocyte antigen DR11. *The Journal of experimental medicine* 189(5), 871-876 (1999).
208. Kobayashi T, Lonchay C, Colau D, Demotte N, Boon T, Van Der Bruggen P. New MAGE-4 antigenic peptide recognized by cytolytic T lymphocytes on HLA-A1 tumor cells. *Tissue Antigens* 62(5), 426-432 (2003).
209. Duffour M-T, Chaux P, Lurquin C, Cornelis G, Boon T, Van Der Bruggen P. A MAGE-A4 peptide presented by HLA-A2 is recognized by cytolytic T lymphocytes. *European Journal of Immunology* 29(10), 3329-3337 (1999).
210. Miyahara Y, Naota H, Wang L *et al*. Determination of Cellularly Processed HLA-A2402-Restricted Novel CTL Epitopes Derived from Two Cancer Germ Line Genes, MAGE-A4 and SAGE. *Clinical Cancer Research* 11(15), 5581 (2005).
211. Zhang Y, Stroobant V, Russo V, Boon T, Van Der Bruggen P. A MAGE-A4 peptide presented by HLA-B37 is recognized on human tumors by cytolytic T lymphocytes. *Tissue Antigens* 60(5), 365-371 (2002).
212. Zorn E, Hercend T. A MAGE-6-encoded peptide is recognized by expanded lymphocytes infiltrating a spontaneously regressing human primary melanoma lesion. *European Journal of Immunology* 29(2), 602-607 (1999).
213. Tanzarella S, Russo V, Lionello I *et al*. Identification of a Promiscuous T-Cell Epitope Encoded by Multiple Members of the MAGE Family. *Cancer Research* 59(11), 2668 (1999).
214. Vantomme V, Boël P, Plaen ED, Boon T, Bruggen PVD. A new tumor-specific antigenic peptide encoded by MAGE-6 is presented to cytolytic T lymphocytes by HLA-Cw16. *Cancer Immunity Archive* 3(1), 17 (2003).
215. Tatsumi T, Kierstead LS, Ranieri E *et al*. MAGE-6 Encodes HLA-DRβ1*0401-presented Epitopes Recognized by CD4+ T Cells from Patients with Melanoma or Renal Cell Carcinoma. *Clinical Cancer Research* 9(3), 947-954 (2003).
216. Oehlrich N, Devitt G, Linnebacher M *et al*. Generation of RAGE-1 and MAGE-9 peptide-specific cytotoxic T-Lymphocyte lines for transfer in patients with renal cell carcinoma. *International Journal of Cancer* 117(2), 256-264 (2005).
217. Huang L-Q, Brasseur F, Serrano A *et al*. Cytolytic T Lymphocytes Recognize an Antigen Encoded by MAGE-A10 on a Human Melanoma. *The Journal of Immunology* 162(11), 6849 (1999).
218. Panelli MC, Bettinotti MP, Lally K *et al*. A Tumor-Infiltrating Lymphocyte from a Melanoma Metastasis with Decreased Expression of Melanoma Differentiation Antigens Recognizes MAGE-12. *The Journal of Immunology* 164(8), 4382 (2000).
219. Anderson LD, Jr., Cook DR, Yamamoto TN, Berger C, Maloney DG, Riddell SR. Identification of MAGE-C1 (CT-7) epitopes for T-cell therapy of multiple myeloma. *Cancer immunology, immunotherapy : CII* 60(7), 985-997 (2011).
220. Nuber N, Curioni-Fontecedro A, Matter C *et al*. Fine analysis of spontaneous MAGE-C1/CT7-specific immunity in melanoma patients. *Proc Natl Acad Sci U S A* 107(34), 15187-15192 (2010).
221. Ma W, Germeau C, Vigneron N *et al*. Two new tumor-specific antigenic peptides encoded by gene MAGE-C2 and presented to cytolytic T lymphocytes by HLA-A2. *International Journal of Cancer* 109(5), 698-702 (2004).
222. Godelaine D, Carrasco J, Brasseur F *et al*. A new tumor-specific antigen encoded by MAGE-C2 and presented to cytolytic T lymphocytes by HLA-B44. *Cancer Immunology, Immunotherapy* 56(6), 753-759 (2007).

223. Ma W, Vigneron N, Chapiro J *et al*. A MAGE-C2 antigenic peptide processed by the immunoproteasome is recognized by cytolytic T cells isolated from a melanoma patient after successful immunotherapy. *International Journal of Cancer* 129(10), 2427-2434 (2011).
224. Wen W, Zhang L, Peng J *et al*. Identification of promiscuous HLA-DR-restricted CD4+ T-cell epitopes on the cancer-testis antigen HCA587. *Cancer Science* 102(8), 1455-1461 (2011).
225. Moreau-Aubry A, Le Guiner S, Labarrière N, Gesnel MC, Jotereau F, Breathnach R. A processed pseudogene codes for a new antigen recognized by a CD8(+) T cell clone on melanoma. *The Journal of experimental medicine* 191(9), 1617-1624 (2000).
226. Jäger E, Chen YT, Drijfhout JW *et al*. Simultaneous humoral and cellular immune response against cancer-testis antigen NY-ESO-1: definition of human histocompatibility leukocyte antigen (HLA)-A2-binding peptide epitopes. *The Journal of experimental medicine* 187(2), 265-270 (1998).
227. Eikawa S, Kakimi K, Isobe M *et al*. Induction of CD8 T-cell responses restricted to multiple HLA class I alleles in a cancer patient by immunization with a 20-mer NY-ESO-1f (NY-ESO-1 91-110) peptide. *International Journal of Cancer* 132(2), 345-354 (2013).
228. Matsuzaki J, Qian F, Luescher I *et al*. Recognition of naturally processed and ovarian cancer reactive CD8+ T cell epitopes within a promiscuous HLA class II T-helper region of NY-ESO-1. *Cancer Immunology, Immunotherapy* 57(8), 1185-1195 (2008).
229. Ebert LM, Liu YC, Clements CS *et al*. A Long, Naturally Presented Immunodominant Epitope from NY-ESO-1 Tumor Antigen: Implications for Cancer Vaccine Design. *Cancer Research* 69(3), 1046-1054 (2009).
230. Knights AJ, Nuber N, Thomson CW *et al*. Modified tumour antigen-encoding mRNA facilitates the analysis of naturally occurring and vaccine-induced CD4 and CD8 T cells in cancer patients. *Cancer Immunology, Immunotherapy* 58(3), 325-338 (2009).
231. Jäger E, Karbach J, Gnjatic S *et al*. Identification of a naturally processed NY-ESO-1 peptide recognized by CD8+ T cells in the context of HLA-B51. *Cancer immunity* 2 12 (2002).
232. Gnjatic S, Nagata Y, Jäger E *et al*. Strategy for monitoring T cell responses to NY-ESO-1 in patients with any HLA class I allele. *Proceedings of the National Academy of Sciences* 97(20), 10917 (2000).
233. Neumann F, Wagner C, Kubuschok B, Stevanovic S, Rammensee H-G, Pfreundschuh M. Identification of an antigenic peptide derived from the cancer-testis antigen NY-ESO-1 binding to a broad range of HLA-DR subtypes. *Cancer Immunology, Immunotherapy* 53(7), 589-599 (2004).
234. Mizote Y, Taniguchi T, Tanaka K *et al*. Three novel NY-ESO-1 epitopes bound to DRB1*0803, DQB1*0401 and DRB1*0901 recognized by CD4 T cells from CHP-NY-ESO-1-vaccinated patients. *Vaccine* 28(32), 5338-5346 (2010).
235. Bioley G, Dousset C, Yeh A *et al*. Vaccination with Recombinant NY-ESO-1 Protein Elicits Immunodominant HLA-DR52b-restricted CD4+ T Cell Responses with a Conserved T Cell Receptor Repertoire. *Clinical Cancer Research* 15(13), 4467 (2009).
236. Chiriva-Internati M, Wang Z, Pochopien S, Salati E, Lim SH. Identification of a sperm protein 17 CTL epitope restricted by HLA-A1. *International Journal of Cancer* 107(5), 863-865 (2003).
237. Ayyoub M, Stevanovic S, Sahin U *et al*. Proteasome-Assisted Identification of a SSX-2-Derived Epitope Recognized by Tumor-Reactive CTL Infiltrating Metastatic Melanoma. *The Journal of Immunology* 168(4), 1717 (2002).
238. Ayyoub M, Hesdorffer CS, Metthez G *et al*. Identification of an SSX-2 Epitope Presented by Dendritic Cells to Circulating Autologous CD4+ T Cells. *The Journal of Immunology* 172(11), 7206-7211 (2004).

239. Neumann F, Kubuschok B, Ertan K *et al*. A peptide epitope derived from the cancer testis antigen HOM-MEL-40/SSX2 capable of inducing CD4+ and CD8+ T-cell as well as B-cell responses. *Cancer Immunology, Immunotherapy* 60(9), 1333 (2011).
240. Ayyoub M, Merlo A, Hesdorffer CS *et al*. Distinct but overlapping T helper epitopes in the 37–58 region of SSX-2. *Clinical Immunology* 114(1), 70-78 (2005).
241. Neumann F, Wagner C, Stevanovic S *et al*. Identification of an HLA-DR-restricted peptide epitope with a promiscuous binding pattern derived from the cancer testis antigen HOM-MEL-40/SSX2. *International Journal of Cancer* 112(4), 661-668 (2004).
242. Ayyoub M, Hesdorffer CS, Montes M *et al*. An immunodominant SSX-2-derived epitope recognized by CD4+ T cells in association with HLA-DR. *The Journal of clinical investigation* 113(8), 1225-1233 (2004).
243. Ayyoub M, Merlo A, Hesdorffer CS *et al*. CD4+ T Cell Responses to SSX-4 in Melanoma Patients. *The Journal of Immunology* 174(8), 5092-5099 (2005).
244. Valmori D, Qian F, Ayyoub M *et al*. Expression of Synovial Sarcoma X (SSX) Antigens in Epithelial Ovarian Cancer and Identification of SSX-4 Epitopes Recognized by CD4+ T Cells. *Clinical Cancer Research* 12(2), 398-404 (2006).
245. Adair SJ, Carr TM, Fink MJ, Slingluff CL, Jr., Hogan KT. The TAG Family of Cancer/Testis Antigens is Widely Expressed in a Variety of Malignancies and Gives Rise to HLA-A2–Restricted Epitopes. *Journal of Immunotherapy* 31(1), (2008).
246. Zhu B, Chen Z, Cheng X *et al*. Identification of HLA-A*0201-restricted Cytotoxic T Lymphocyte Epitope from TRAG-3 Antigen. *Clinical Cancer Research* 9(5), 1850 (2003).
247. Lupetti R, Pisarra P, Verrecchia A *et al*. Translation of a retained intron in tyrosinase-related protein (TRP) 2 mRNA generates a new cytotoxic T lymphocyte (CTL)-defined and shared human melanoma antigen not expressed in normal cells of the melanocytic lineage. *The Journal of experimental medicine* 188(6), 1005-1016 (1998).
248. Ohue Y, Eikawa S, Okazaki N *et al*. Spontaneous antibody, and CD4 and CD8 T-cell responses against XAGE-1b (GAGED2a) in non-small cell lung cancer patients. *International Journal of Cancer* 131(5), E649-E658 (2012).
249. Shimono M, Uenaka A, Noguchi Y *et al*. Identification of DR9-restricted XAGE antigen on lung adenocarcinoma recognized by autologous CD4 T-cells. *Int J Oncol* 30(4), 835-840 (2007).
